# Modeling and performance evaluation of hybrid photovoltaic thermal, wind, and battery microgrids using optimization and dynamic simulation

**DOI:** 10.1038/s41598-025-95149-w

**Published:** 2025-04-04

**Authors:** Etoju Jacob, Hooman Farzaneh

**Affiliations:** 1 Interdisciplinary Graduate School of Engineering Sciences, Kyushu University, Fukuoka, 816-8580 Japan; 2 Transdisciplinary Research and Education Center for Green Technologies, Kyushu University, Fukuoka, Japan

**Keywords:** Photovoltaic thermal (PV/T), Microgrid, Off-grid, Battery-life, Simulation, Optimization, Energy infrastructure, Energy grids and networks

## Abstract

This study aims to comprehensively develop a modeling framework to evaluate the dynamic performance of a photovoltaic/thermal (PV/T) system integrated with a hybrid off-grid microgrid. The advancements made by this research in investigating the optimal design of the PV/T system and dynamic performance assessment of the proposed hybrid microgrid are twofold. First, a nonlinear mathematical problem is formulated to determine the optimal system design that maximizes power, taking into account the thermo-electrical constraints. Secondly, the research highlights the development of a component mask with a user-defined functionality in MATLAB/Simulink using the optimal design parameters obtained from the optimization model. The developed PV/T component is then integrated with a wind turbine/ battery system. The resultant integrated energy system is then compared with a conventional PV/wind/battery microgrid system based on a 72-hour simulation. The outcomes showed that under cloudy, rainy, sunny, and windy conditions, the extra cumulative electricity generation from the PV/T system-based microgrid is 2.12, 2.74, 1.72, and 0.31% compared to the PV system-based microgrid. Additionally, improved battery system operation of nearly 1.8*Wh* was realized, signifying PV/T contribution to efficient microgrid operation.

**Supplementary Information:**

The online version contains supplementary material available at 10.1038/s41598-025-95149-w.

## Introduction

### Background

Energy prices have risen dramatically due to the current global energy crisis, and 90% of the increase in electricity prices in 2022 may be attributed to the cost of fossil fuels^1^. As a result, stakeholders have been forced to re-evaluate their policy priorities, placing the most focus on energy security. The current crisis may ultimately be considered pivotal in the fight for both energy security and emissions reductions. This is because most measures are also needed to accomplish the targets set in the international climate agreements. Consequently, to achieve secure energy transitions, there must be a strong focus on increasing investment in clean energy technology and raising energy efficiency.

As a clean solution, solar technologies (thermal collectors and PV) are frequently used to generate thermal energy and electrical energy, respectively. However, the primary impediments to the widespread usage of PV systems are their high initial investment costs and very poor conversion efficiencies, ranging from 6 to 25% depending on the solar cell type^2^. Several factors affect PV module energy conversion effectiveness. These include cell material (from degradation to contact stability), auxiliary devices (from controllers to batteries), and environmental conditions. Notably, the PV cell temperature is mainly influenced by the latter, which significantly impacts the conversion efficiency of PV modules^3^. According to studies, monocrystalline (c-Si), polycrystalline (pc-Si), and amorphous (a-Si) silicon cells experience a reduction in electrical conversion efficiency of 0.45%, 0.45%, and 0.25%, respectively, with each degree of temperature increase^4^.

PV modules need to be cooled to obtain greater conversion efficiency. As a result, active and passive cooling solutions utilizing various media have been suggested for top or back surface cooling. The photovoltaic thermal (PV/T) system is the most contemporary technology that simultaneously generates electricity and heat using the solar thermal collector (STC) and PV technologies. Compared to a conventional PV or STC with the same footprint, PV/T modules are more efficient. The electrical output is improved by chilling the PV cells with a medium such as air, water, nanofluids, bi-fluids, refrigerants, heat pipes, and phase change materials (PCM)^5^. At the same time, the heat from the cooling fluid can be used for thermal applications.

To date, effort has been put into developing PV/T systems mainly for performance analysis, neglecting their synergistic role in microgrids. Moreover, reliance on a single technology brings about reliability issues. In particular, hybridizing PV/T systems with other energy sources improves system efficiency and energy utilization. The PV/T also covers thermal needs, which are normally met by the grid, deeming it cost-effective. Additionally, PV/T modules have higher electrical energy yields. This positively impacts battery cycling when demand increases.

### Literature review

#### Theoretical and experimental research on the performance analysis of PV/T systems

The literature on PV/T systems is not as substantial as that on conventional PV and STCs. However, investigations on PV/T systems using numerical models, simulations, and tests to assess their performance have been suggested. The effectiveness of various active air-based PV/T system configurations and their effects on buildings were theoretically and experimentally examined by Tomar et al.^6^. Their analytical model produced an average electrical and thermal efficiency of 12.65% and 32.77% for the PV with a duct incorporated, as opposed to 12.7% and 25.44% without the duct.

Water-based PV/T systems have received greater attention recently since they attain higher overall efficiency than air systems due to the higher water heat capacity and density^7^. A quasi-steady state 1-D water-based PV/T model based on fin heat transfer for combined domestic cooling and water heating was examined by Zarei et al.^8^. With water mass flow rates varying from 0.011 kg/s to 0.03 kg/s, the results showed an improved overall efficiency of 66.7–75.8%. Bhattarai et al.^9^ proposed a 1-D mathematical model for the dynamic states of a sheet and tube PV/T and evaluated the performance in simulations and experiments compared to a standard STC. The PV/T was determined to be 13.69% electrically efficient, with the thermal efficiency of the STC around 18% higher. However, the PV/T saved 16% more primary energy than the STC. A 1-D numerical model of a PV/T plant was presented and examined by El Fouas et al.^10^ in a Mediterranean city. The system produced 0.83 kWh/m^2^ of electrical energy and 0.53 kWh/m^2^ of thermal energy per day.

Further research into different designs in PV/T modules reveals the potential for performance enhancement. Colombini et al.^11^ explored a dynamic 2-D PV/T model with a roll-bond absorber and examined the harp, serpentine, and spiral absorber design layouts. The serpentine structure had the maximum thermal efficiency (46%), although it suffered from a pressure drop. Kaewchoothong et al.^12^ examined the heat dissipation and working fluid flow properties in a ribbed parallel channel water-based PV/T module. The continuous V-shaped rib offered increased electrical efficiency between 0.8% and 1.5% compared to a PV panel. Based on the notion of field synergy, a water-based PV/T with a shark dorsal fin-type saw teeth channel was created by Shen et al.^13^ to maximize system performance. The average cell temperature was around 6 °C lower in the unique channel than in the traditional smooth channel. A major overall impact is also produced by the design of glazed or unglazed PV/T modules. In the works of Maadi et al.^4^, a 3-D coupled thermal-optical model was created to assess the operation of a glazed PV/T module. With the glazing, a thermal yield enhancement of nearly 12% more than that of the unglazed configuration was achieved. Higher energy efficiency was also found in glazed modules in a related work by Yazdanifard et al.^14^ on a water-based sheet and tube PV/T. To this end, glazed PV/T(s) are superior to unglazed ones if the goal is to increase thermal output and overall efficiency; nevertheless, larger glass covers reduce electrical efficiency.

#### Research on the integration of PV/T systems with hybrid microgrids

Accordingly, the prior PV/T system literature focuses on performance assessment based on system efficiency, with some consideration of parametric analysis. However, in energy systems, reliability is essential and cannot be achieved by having a dedicated source based on intermittent energy resources^15^. Since the current study entails PV/T integration into a hybrid microgrid, some recent work on microgrid systems with PV/T collectors integrated into buildings to assess their operations is also discussed and presented in Table 1.

**Table 1:** Summary of works incorporating the PV/T with hybrid microgrids.

Ref.	System	Comprehensive model		Approach	Microgrid analysis	Limitations
		Electrical	Thermal			
^16^	PV/T-wind-thermal storage	x	x	Techno-economic	x	Focused on the thermal storage model and economic analysis.
^17^	BiPV/T-thermal storage	x	x	Numerical, experimental	✓	No comprehensive PV/T model.
^18^	PV/T- battery-thermochemical sorption storage	✓	✓	Numerical	x	No operational simulation.
^19^	CPV/T-Fuel cell-Electrolyzer	x	✓	Numerical	x	No dynamic simulation and generally focused on a parametric analysis.
^20^	PV/T-PTSC-Electrolyzer-thermal storage	x	✓	Numerical	x	The analysis of the hybrid system is mainly focused on energy and exergy.
^21^	PV/T-battery-thermal storage	x	x	Numerical, experimental	✓	No comprehensive numerical model was presented.
This study	PV/T-wind-battery	✓	✓	Dynamic simulation	✓	No experimental setup

#### Impact of PV/T system on battery life and performance

An effective energy storage system, often a battery, is necessary for HRES to function well. Operating conditions, working atmosphere, and battery charging technique affect how well a battery performs^22^. Batteries typically age due to calendaring, which is heavily affected by time and temperature, and cycling, which is influenced by temperature, charge/discharge rate, and energy throughput (DoD)^23^. According to an experimental analysis using a lithium iron phosphate battery, cycle life decreases more rapidly as the charge current rate increases^24^. Additionally, the heat produced during charging/discharging and the surroundings significantly impacts battery temperature, facilitating mechanical stress. In particular, deep discharge, overcharge, and partial cycling^25^ induce sulphation, acid stratification, impedance development^26^, and active-mass loss in lead-acid batteries. Due to these degrading processes, batteries prematurely lose capacity over time. To this end, Jakov et al. showed the impact of component capacity loss on system performance and energy reliability^25^. Contrarily, battery performance can be enhanced by designing the internal material structure optimally and using it efficiently. With the latter, Saeed et al. demonstrated that pulse charging, as opposed to constant voltage charging, can extend battery life by more than 18%^27^. Alphonce and Farzaneh realized an annual potential of 858*kWh* storage capacity gain in battery coupled with a flywheel system^28^. The significance of a supercapacitor on battery longevity in a hybrid power system was investigated by Roy et al.^29^. They observed a two-fold increment in battery lifetime when both were integrated. Furthermore, a forecast-based microgrid operation strategy was introduced in^30^ that limits SoC by storing only the energy required at night in order to increase battery lifetime. However, subsequent supply forecasts are not considered. In light of the review, the importance and impact of overall system operation on storage dynamics is highlighted. Moreover, no studies have covered the enhancement of battery performance due to PV/T microgrid operation. This study, therefore, provides valuable insights into the performance through quantification in terms of SoC and voltage accumulation rate. The study highlights the dependence of battery performance on the choice of microgrid operation strategy.

### Research gap and paper contributions

Comprehensive research on modeling, performance evaluation, and integration of PV/T collectors to increase system efficiency has been conducted; however, PV/T systems are still not well established academically or commercially. Although previous research works have already addressed various theoretical and experimental methods for optimal design and configuration of the PV/T systems. Regardless, their key role in hybrid microgrids of improving system effectiveness and reliability under stochastic conditions has not been paid much attention, and the current studies conducted in this field are still insufficient, as presented in Table 1.

To fill the above gaps, this research presents a comprehensive modeling framework that can be used for the dynamic performance analysis of a sheet and tube-based PV/T, wind, and battery microgrid. The sheet and tube arrangement are selected because it offers the best chance of success in practical applications. The proposed modeling framework has two parts:

#### Optimal design of the PV/T

Most thermo-physical and geometrical properties, such as electrical parameters (diode saturation currents, shunt, and series resistances), flow rate, thermal conductivities, and thicknesses, are not usually specified by manufacturers. This forms the basis for optimally finding the key operational PV/T features based on the thermal-electrical modeling approach. This is achieved through the PV/T system output power maximization, which is subject to satisfying the thermal-electrical constraints. The determined electrical parameters and the flow rate are then used for the PV/T performance evaluation through dynamic simulation in an off-grid microgrid configuration.

#### Dynamic power simulation of the microgrid with the PV/T

In this study, the microgrid model is developed in MATLAB/Simulink. In Simulink, the simulation properties for different tasks include system-level behavior, average value, and full switching. Unlike in^19,20^, and^21^, where they deal with higher-level energy flow and efficiencies, within the framework of this study, the split-second fast dynamic transient behavior of the system states is considered. Therefore, the model design considers the “Powergui block” to facilitate the choice among three system-solving approaches, namely: (1) continuous, which uses a variable-step solver; (2) discrete, which uses a fixed-step solver; and (3) phasor, which excludes physical system dynamics^31^.

Power electronic building blocks are required in model design, of which the PV/T system is absent. Therefore, the PV/T system component block is developed based on the optimal design features (electrical parameters and flow rate). It is then integrated with a DC microgrid and dynamically simulated in MATLAB/Simulink to evaluate its performance. The microgrid comprises a water-based PV/T module, wind turbine system, battery, and a dynamic load, all conventionally controlled as presented in^15^. A 72-hour operational simulation was conducted to compare the energy enhancement of a PV/T-based microgrid against a conventional PV-based microgrid, using meteorological conditions from Fukuoka, Japan.

Overall, the combination of two system models (electrical and thermal) through optimization and a dynamic simulation considering system state dynamics contributes to improving system effectiveness and reliability in microgrids under stochastic conditions, which has not yet been studied.

Therefore, the main objectives of this study are as follows:

(i) A comprehensive 1D steady-state model is developed, which integrates the five-parameter PV model with a thermal-resistance network model to find the optimal design of the PV/T system through power maximization.
(ii) A new component mask with user-defined functionality is developed in Matlab-Simulink, using the design configuration of the sheet and tube-based parallel flow PV/T system.
(iii) The developed PV/T component is then integrated with the wind turbine/battery/dynamic load and compared with a conventional PV/wind/battery microgrid system based on a 72-*hour* simulation to assess how the suggested microgrid performs dynamically and enhances energy generation.
(iv) Furthermore, the role of the PV/T system (charging technology) in managing the battery cycling through improving the battery performance is quantified with SoC and voltage accumulation rate. This is not covered in most studies.

The remaining sections are structured as follows: Section “System description” covers the configuration and physical system components. The mathematical modeling and foundation of the microgrid components are detailed in Section “Model development”, with emphasis on the PV/T system, whereas Section “Results and discussion” presents the findings and discussion. Lastly, Section “Conclusions” discusses the conclusion.

## System description

**Fig. 1:**
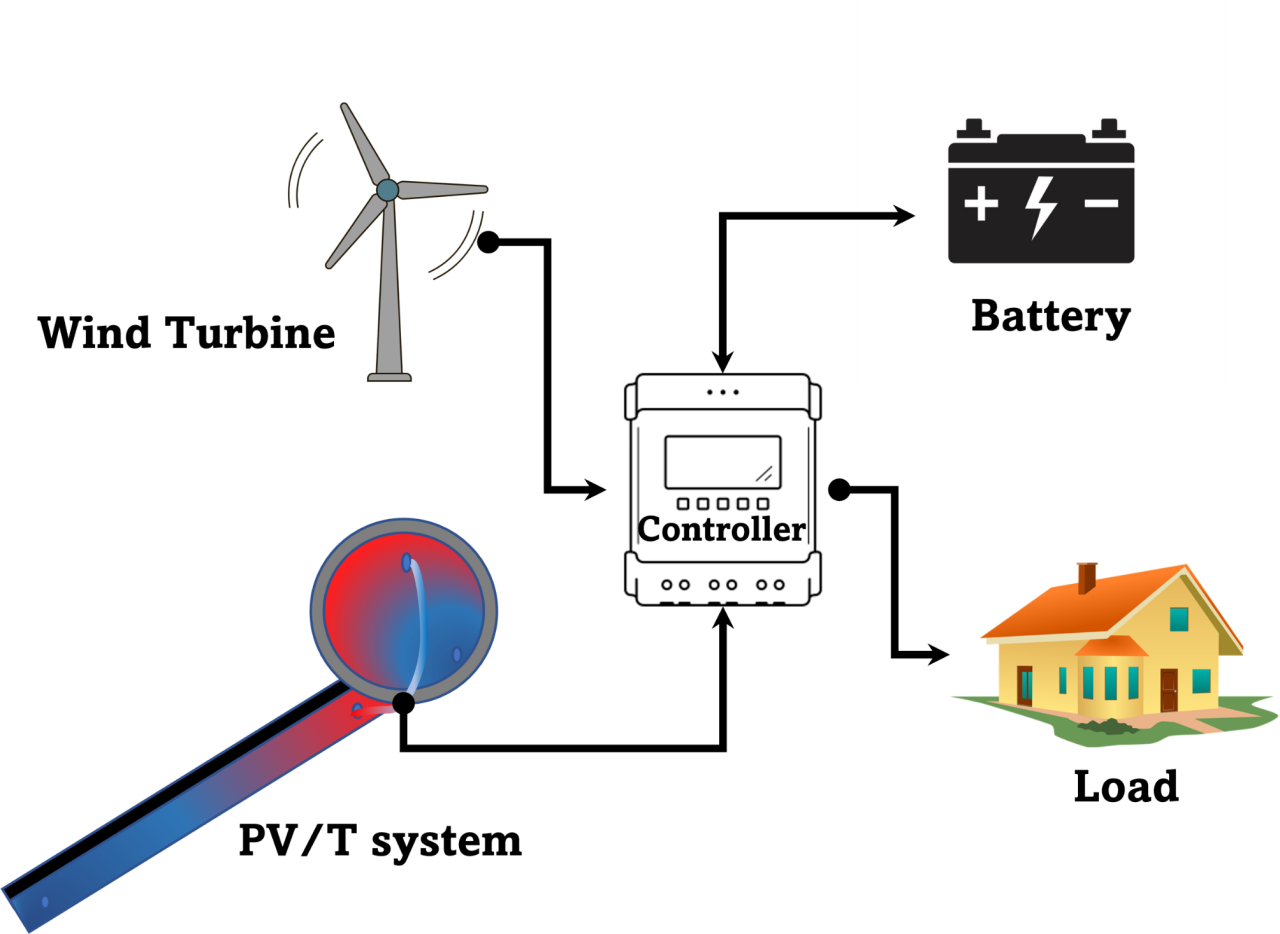
Microgrid system configuration.

The hybrid DC microgrid system generally consists of a parallel flow water-based PV/T module, battery, and wind turbine system as the main generation components. The proposed configuration is shown in Fig. 1.

**Fig. 2:**
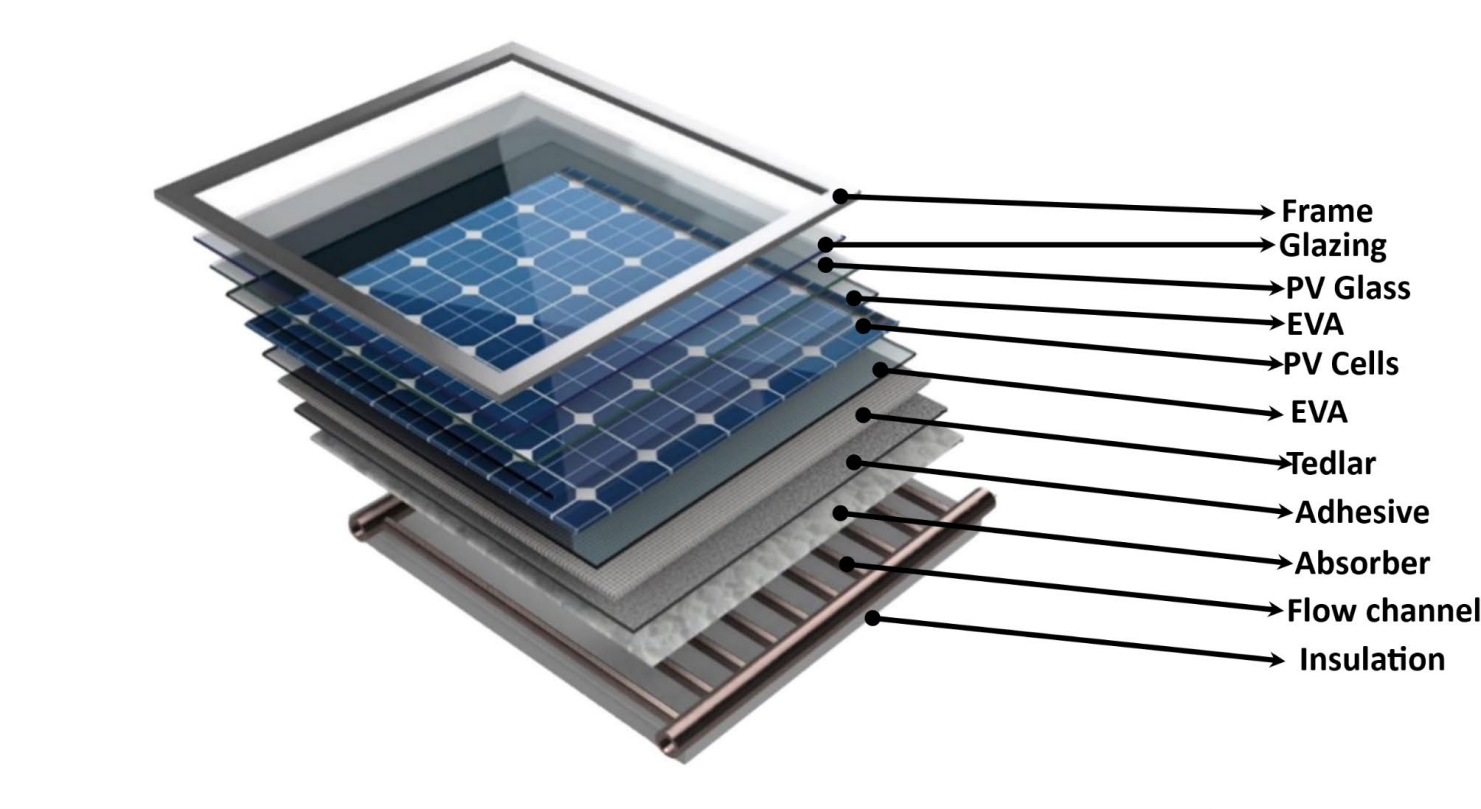
Visualization of the PV/T component^5^.

The proposed PV/T is shown in Fig. 2, and the parameters are reported in Tables 2, 3 and 4.

**Table 2:** PV/T module electrical specifications used in the simulation^10,32^.

Parameter	Value
$\:{\varvec{P}}_{\varvec{m}\varvec{p}}$	250 W^1^
$\:{\varvec{V}}_{\varvec{m}\varvec{p}}$	30.7 V
$\:{\varvec{I}}_{\varvec{m}\varvec{p}}$	8.15 A
$\:{\varvec{I}}_{\varvec{s}\varvec{c}}$	8.55 A
$\:{\varvec{V}}_{\varvec{o}\varvec{c}}$	38.5 V
$\:{\varvec{\beta\:}}_{\varvec{c}}$	− 0.44%/K
$\:{\varvec{\mu\:}}_{\varvec{s}\varvec{c}}$	0.048%/K
$\:{\varvec{N}}_{\varvec{s}}$	60
$\:\varvec{P}\varvec{F}$	0.9
$\:{\varvec{\eta\:}}_{\varvec{S}\varvec{T}\varvec{C}}$	15.4%

^1^ Dimensions: 2094 × 1038 × 35 mm.

**Table 3:** PV/T geometrical, thermal, and material properties used in the simulation.

Property	Glass	PV^2^	Adhesive	Absorber	Bond	Tube	Insulation	Ref.
Absorptivity, $\:\varvec{\alpha\:}$ (–)	–	0.93	–	–	–	–	–	^33^
Transmissivity, $\:\varvec{\tau\:}$ (–)	0.92	–	–	–	–	–	–	^10^
Emissivity, $\:\varvec{\epsilon\:}$ (–)	–	0.9	–	–	–	–	–	^33^
Thermal conductivity, $\:\varvec{k}$ (W/mK)	–	100	0.85	310	310	310	0.03	^32^
Thickness, $\:\varvec{x}$ (m)	–	1.4e−3	5e−5	2e−4	9e−4	12e−3	5e−2	^32,34^
External Diameter, $\:{\varvec{D}}_{\varvec{o}}$ (m)	–	–	–	–	–	0.01	–	^32^
Risers, $\:\varvec{n}$ (–)	–	–	–	–	–	10	–	^34^
Length,$\:\:\varvec{L}$ (m)	–	–	–	–	–	2	–	^14^
Width / Spacing, $\:\varvec{w}$ (m)	–	–	–	–	0.01	0.1	–	^14^

^2^ PV laminate includes the ARC, tedlar and 2 EVA layers.

**Table 4:** Other PV/T accessories specifications.

Property	Tank	Exchanger	Ref
Effectiveness, $\:{\varvec{\epsilon\:}}_{\varvec{H}}$ (–)	–	0.4	^32^
Heat loss coefficient, $\:\varvec{U}$ (W/m^2^*K)*	0.8	–	^35^
Inner surface area, $\:\varvec{S}\varvec{A}$ (m^2^*)*	1.8	–	

The PV/T has two main units, the PV and solar collector. The PV/T is an indirect parallel flow type where the working fluid (water) is disseminated to a heat exchanger in the thermal storage. The fluid flow velocity, number of glass covers, type of cell, and thermophysical parameters like geometry and tube diameter significantly impact the system efficiency. The PV layer/sandwich comprises transparent glass, two encapsulants (ethyl vinyl acetate-EVA), and tedlar—all combined to protect the PV cells from UV radiation and humidity^36^. A copper sheet absorber with riser tubes is attached beneath to harness the accumulated thermal energy. The absorber’s surface is covered with a film of adhesive with satisfactory heat conduction properties^37^.

The rest of the microgrid includes a horizontal-axis wind turbine, whose output power depends on the blade span, wind speed, and power coefficient^38^. For power backup, a battery is essential for voltage stability, energy balance, and reducing the cost of electricity if grid-tied. The wind turbine system and battery specifications are shown in Tables 5, and 6, respectively.

**Table 5:** Wind turbine system component specifications^15^.

Parameter	Specification
Rated power	100 W
Diameter	0.58 m
Tip speed ratio	4
Rated wind speed	11.4 (m/s)
Power coefficient	0.48

**Table 6:** V30-800 Lead-acid battery specifications and charging characteristics^39^.

Parameter	Value
Fully charge voltage	14 V
20 h capacity	30 Ah
RC (min)	55
CCA	280
Charging current	2–10 A
Charging voltage	14.4–14.9 V
Float voltage	13.5–13.8 V

## Model development

The modeling approach consists of two phases. The first phase involves optimally designing the PV/T system, with a focus on maximizing its electrical power while fulfilling both electrical and thermal design constraints. In the second phase, the optimized PV/T system is integrated with a wind turbine and battery in a microgrid configuration. This microgrid configuration is then simulated in Simulink to evaluate the system’s dynamic performance in detail.

### PV/T optimal design model

The PV/T power output is influenced by the equivalent circuit parameters and cell temperature under different operating conditions. Therefore, a nonlinear optimization problem is formulated using the ideal five-parameter single-diode PV model derived from the I-V characteristic^40^. The objective function includes maximizing the electrical power and is as follows:

1 $$\:\underset{\{{T}_{c},\:\:\dot{m}\:\in\mathbb{\:}\mathbb{R}\}}{max}\:\mathcal{\ell}\:\left({{T}_{c}}^{t}\right({\dot{m}}^{t}\left)\right)$$

where, $\:\mathcal{\ell}\mathcal{\:}\left({{T}_{c}}^{t}\right({\dot{m}}^{t}\left)\right)$ is the objective function.

$$\:\mathcal{\ell}\mathcal{\:}\left({{T}_{c}}^{t}\right({\dot{m}}^{t}\left)\right)=\left({{I}_{L\left({T}_{c}\right)}}^{t}-{{I}_{o\left({T}_{c}\right)}}^{t}\:\left[exp\left(\frac{({V}^{t}\:+\:{I}^{t}\:{{R}_{s}}^{t})}{{{a}_{{T}_{c}}}^{t}}\right)-1\right]-\frac{{V}^{t}\:+\:{I}^{t}\:{{R}_{s}}^{t}}{{{R}_{sh}}^{t}}\right)\:{V}^{t}\:,\:\forall\:t\in\:[1,\:2\dots\:,N]$$

The objective function (1) is maximized subject to the following electrical system parameter constraints (2)–(7):

2 $$\:{{I}_{L\left({T}_{c}\right)}}^{t}=\frac{{S}^{t}}{{S}_{ref}}\left[{I}_{L,ref}+{\mu\:}_{L,sc}\left({{T}_{c}}^{t}-{T}_{c,\text{\:ref\:}}\right)\right]$$

3 $$\:{{I}_{o\left({T}_{c}\right)}}^{t}\:={{I}_{o,\text{\:ref\:}}\:\left(\frac{{{T}_{c}}^{t}}{{T}_{c,\text{\:ref\:}}}\right)}^{3}exp\left({\left.\frac{1.12 \, eV}{{k}^{*}T}\right|}_{{T}_{c,\text{\:ref\:}}}-{\left.\frac{1.12 \, eV\left(1-0.0002677({{T}_{c}}^{t}-{T}_{c,ref})\right)}{{k}^{*}T}\right|}_{{{T}_{c}}^{t}}\right)$$

4 $$\:{{a}_{{T}_{c}}}^{t}=\frac{{{T}_{c}}^{t}}{{T}_{c,ref}}\left(\frac{{n}^{d}{k}^{b}{T}_{c,ref}{N}_{s}}{q}\right)$$

5 $$\:{{R}_{s}}^{t}={R}_{s,ref}$$

6 $$\:{{R}_{sh}}^{t}=\frac{{S}_{ref}}{{S}^{t}}\:{R}_{sh,ref}$$

7 $$\:{S}^{t}=\frac{{G}^{t}}{{G}_{ref}}\:{S}_{ref}$$

The objective function (1) aims to find the design conditions and working fluid flow rate that achieve maximum power from the PV/T system, taking the thermo-electrical properties into account. The variables in the objective function are related to the PV power output model. Equality constraints (2)–(7) are discussed as follows: (2) accounts for the photocurrent dependence on cell temperature and absorbed irradiation; (3) caters to the diode saturation current dependence on cell temperature and silicon band-gap energy; (4) considers the influence of cell temperature on the modified diode ideality factor; (5) relates the series resistance; and (6) caters for the dependence of shunt resistance on absorbed irradiation while (7) relates the absorbed to the incident irradiation. The optimal parameter extraction at reference conditions is presented in Table 7.

**Table 7:** Extracted PV/T electrical parameters at standard test conditions.

Parameter	Value
$\:\varvec{a}$	1.193
$\:{\varvec{I}}_{\varvec{L}}$	8.555 A
$\:{\varvec{I}}_{\varvec{o}}$	8.2491e−14 A
$\:{\varvec{R}}_{\varvec{s}}$	0.495 Ω
$\:{\varvec{R}}_{\varvec{s}\varvec{h}}$	830.568 Ω

The objective function (1) also depicts dependence on the cell temperature, $\:{{T}_{c}}^{t}$ and this necessitates the inclusion of the thermal model as a constraint in order to estimate $\:{{T}_{c}}^{t}$. The cell temperature is also highly dependent on the fluid mass flow rate, $\:{\dot{m}}^{t}$ and the correlation is derived through heat exchanger pinch point analysis. A heat exchanger’s maximum heat transfer capacity is restricted by $\:\varDelta\:{T}_{min}$, the lowest temperature differential that can exist between the hot and cold streams^41^. For the PV/T, $\:\varDelta\:{T}_{min}$ is approximated at 0.5 °C from^10,32^, which is used in the cell temperature-fluid mass flow rate relation below:

8 $$\:\varDelta\:{T}_{min}=\:{{T}_{c}}^{t}-\:{{T}_{f}}^{t}$$

9 $$\:\left.{{T}_{f}}^{t}=0.5{\left(T\right.}_{co-in}+{{T}_{co-out}}^{t}\right)$$

10 $$\:{{Q}_{12}}^{t}={\dot{m}}^{t}{{c}_{f}}^{t}\left({{T}_{co-out}}^{t}-{T}_{co-in}\right)$$

From (8)–(10) yields the relation;

11 $$\:{{T}_{c}}^{t}=\frac{{{Q}_{12}}^{t}}{{\dot{m}}^{t}{{c}_{f}}^{t}}+\:\varDelta\:{T}_{min}+{T}_{co-in}$$

**Fig. 3:**
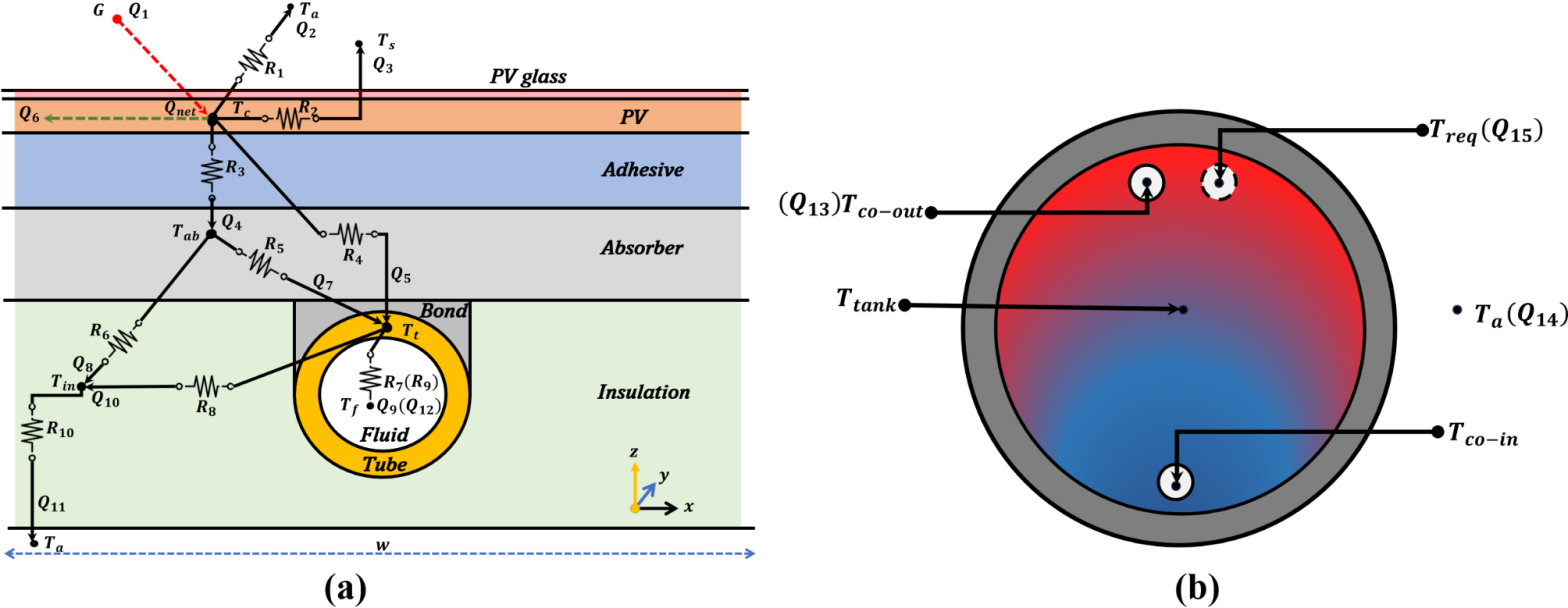
PV/T system components: (**a**) cross-section at one riser, (**b**) tank cross-section.

As highlighted, the thermal model is required to estimate the cell temperature and is established based on steady-state energy balances between the different components, as shown in Fig. 3a. The following assumptions are considered in its development:

(i) 1-D steady-state heat transfer approach, neglecting heat capacity effects.
(ii) Perfect bonding between elements, hence isothermal temperature distribution.
(iii) Fully developed laminar flow in risers, with total flow equally divided.
(iv) Uniform heat transfer throughout the collector length with adiabatic sides.
(v) The impact of connecting headers on temperature distribution is disregarded because they only cover a small portion of the collector^33^.
(vi) One-tube section is used for the model development, as in^4^.
(vii) There is no consideration of friction in the risers; hence, there is no pressure drop analysis.

The heat transfer mechanism in a riser section of width, $\:w,$ and length, $\:L$ in Fig. 3a is as follows:

12 $$\:{{Q}_{net}}^{t}={{Q}_{1}}^{t}-\:{{Q}_{2}}^{t}-\:{{Q}_{3}}^{t}-\:{{Q}_{6}}^{t}=\:{{Q}_{4}}^{t}+{{Q}_{5}}^{t}\:$$

13 $$\:{{Q}_{4}}^{t}={{Q}_{7}}^{t}+\:{{Q}_{8}}^{t}$$

14 $$\:{{Q}_{5}}^{t}+{{Q}_{7}}^{t}\:={{Q}_{9}}^{t}+\:{{Q}_{10}}^{t}$$

15 $$\:{{Q}_{8}}^{t}+{{Q}_{10}}^{t}\:={{Q}_{11}}^{t}$$

16 $$\:{{Q}_{9}}^{t}\:={{Q}_{12}}^{t}$$

17 $$\:{{Q}_{13}}^{t}={{Q}_{14}}^{t}+\:{{Q}_{15}}^{t}$$

18 $$\:{{T}_{c}}^{t}\:,{{T}_{ab}}^{t}\:,{{T}_{t}}^{t}\:,{{T}_{ins}}^{t}\:,{{T}_{f}}^{t}\:,{{T}_{co-out}}^{t}\:,{{T}_{tank}}^{t}\:\ge\:{{T}_{a}}^{t}\:$$

19 $$\:{\dot{m}}^{t}>0$$

20 $$\:{T}_{co-in}\ge\:298$$

21 $$\:{Re}^{t}<2300$$

The flux exchanges, heat transfer coefficients derived from^14^, and other correlations between the different components are defined in Table 8.

**Table 8:** Flux exchanges, heat transfer coefficients, and other correlations between the different components.

Energy Flux	Heat transfer coefficient	Other definitions
$\:{{\varvec{Q}}_{1}}^{\varvec{t}}=\:{\varvec{\alpha\:}\varvec{\tau\:}}_{\text{}}\left(\varvec{w}\varvec{L}\right){\varvec{G}}^{\varvec{t}}$ $\:{{\varvec{Q}}_{2}}^{\varvec{t}}=\:{{\varvec{h}}_{1}}^{\varvec{t}}\left(\varvec{w}\varvec{L}\right)(\:{{\varvec{T}}_{\varvec{c}}}^{\varvec{t}}\:-{{\varvec{T}}_{\varvec{a}}}^{\varvec{t}})$ $\:{{\varvec{Q}}_{3}}^{\varvec{t}}=\:{{\varvec{h}}_{2}}^{\varvec{t}}\left(\varvec{w}\varvec{L}\right)(\:{{\varvec{T}}_{\varvec{c}}}^{\varvec{t}}\:-{{\varvec{T}}_{\varvec{s}}}^{\varvec{t}})$ $\:{{\varvec{Q}}_{4}}^{\varvec{t}}=\:{\varvec{h}}_{3}\left(\varvec{w}\varvec{L}\right)(\:{{\varvec{T}}_{\varvec{c}}}^{\varvec{t}}\:-{{\varvec{T}}_{\varvec{a}\varvec{b}}}^{\varvec{t}})$ $\:{{\varvec{Q}}_{5}}^{\varvec{t}}=\:{\varvec{h}}_{4}\left(\varvec{w}\varvec{L}\right)({{\varvec{T}}_{\varvec{c}}}^{\varvec{t}}\:-{{\varvec{T}}_{\varvec{t}}}^{\varvec{t}})$ $\:{{\varvec{Q}}_{6}}^{\varvec{t}}=\:\left(\varvec{P}\varvec{F}\right)\left(\varvec{w}\varvec{L}\right){{\varvec{\eta\:}}_{\varvec{e}\varvec{l}\varvec{e}}}^{\varvec{t}}\:{\varvec{\tau\:}}_{\varvec{g}}{\varvec{G}}^{\varvec{t}}$ $\:{{\varvec{Q}}_{7}}^{\varvec{t}}=\:{\varvec{h}}_{5}\left(\varvec{w}\varvec{L}\right)\left(\:{{\varvec{T}}_{\varvec{a}\varvec{b}}}^{\varvec{t}}\:-{{\varvec{T}}_{\varvec{t}}}^{\varvec{t}}\right)$ $\:{{\varvec{Q}}_{8}}^{\varvec{t}}=\:{\varvec{h}}_{6}\left(\varvec{w}\varvec{L}\right)\left(\:{{\varvec{T}}_{\varvec{a}\varvec{b}}}^{\varvec{t}}\:-{{\varvec{T}}_{\varvec{i}\varvec{n}\varvec{s}}}^{\varvec{t}}\right)$ $\:{{\varvec{Q}}_{9}}^{\varvec{t}}=\:{\varvec{h}}_{7}\left(\varvec{w}\varvec{L}\right)\left(\:{{\varvec{T}}_{\varvec{t}}}^{\varvec{t}}\:-{{\varvec{T}}_{\varvec{f}}}^{\varvec{t}}\right)$ $\:{{\varvec{Q}}_{10}}^{\varvec{t}}=\:{\varvec{h}}_{8}\left(\varvec{w}\varvec{L}\right)\left(\:{{\varvec{T}}_{\varvec{t}}}^{\varvec{t}}\:-{{\varvec{T}}_{\varvec{i}\varvec{n}\varvec{s}}}^{\varvec{t}}\right)$ $\:{{\varvec{Q}}_{11}}^{\varvec{t}}=\:{{\varvec{h}}_{10}}^{\varvec{t}}\left(\varvec{w}\varvec{L}\right)\left(\:{{\varvec{T}}_{\varvec{i}\varvec{n}\varvec{s}}}^{\varvec{t}}\:-{{\varvec{T}}_{\varvec{a}}}^{\varvec{t}}\right)$ $\:{{\varvec{Q}}_{12}}^{\varvec{t}}={\dot{\varvec{m}}}^{\varvec{t}}{{\varvec{c}}_{\varvec{f}}}^{\varvec{t}}\left({{\varvec{T}}_{\varvec{c}\varvec{o}-\varvec{o}\varvec{u}\varvec{t}}}^{\varvec{t}}-{\varvec{T}}_{\varvec{c}\varvec{o}-\varvec{i}\varvec{n}}\right)$ $\:{{\varvec{Q}}_{13}}^{\varvec{t}}={\dot{\varvec{m}}}^{\varvec{t}}\varvec{n}{{\varvec{c}}_{\varvec{f}}}^{\varvec{t}}{\varvec{\epsilon\:}}_{\varvec{H}}\left({{\varvec{T}}_{\varvec{c}\varvec{o}-\varvec{o}\varvec{u}\varvec{t}}}^{\varvec{t}}-{{\varvec{T}}_{\varvec{t}\varvec{a}\varvec{n}\varvec{k}}}^{\varvec{t}}\right)$ $\:{{\varvec{Q}}_{14}}^{\varvec{t}}=\:{\varvec{U}}_{\varvec{t}\varvec{a}\varvec{n}\varvec{k}}\left(\varvec{S}\varvec{A}\right)(\:{{\varvec{T}}_{\varvec{t}\varvec{a}\varvec{n}\varvec{k}}}^{\varvec{t}}\:-{{\varvec{T}}_{\varvec{a}}}^{\varvec{t}})$ $\:{{\varvec{Q}}_{15}}^{\varvec{t}}={{{\dot{\varvec{m}}}_{\varvec{l}}}^{\varvec{t}}{\varvec{c}}_{\varvec{f}}}^{\varvec{t}}\left({{\varvec{T}}_{\varvec{t}\varvec{a}\varvec{n}\varvec{k}}}^{\varvec{t}}-{{\varvec{T}}_{\varvec{r}\varvec{e}\varvec{q}}}^{\varvec{t}}\right)$	$$\:{{h}_{1}}^{t}=\:\left\{\begin{array}{c}5.7+3.8{{v}_{w}}^{t}\:\:\:\:\:\:\:\:for\:{{v}_{w}}^{t}<5 \, m/s\:\\\:6.47+{{{v}_{w}}^{t}}^{0.78}\:\:for\:{{v}_{w}}^{t}>5 \, m/s\end{array}\right.$$ ^4^ $\:{{h}_{2}}^{t}={\epsilon\:}_{c}\sigma\:\left({{{T}_{c}}^{t}}^{2}+{{{T}_{s}}^{t}}^{2}\right)\left({{T}_{c}}^{t}+{{T}_{s}}^{t}\right)$ ^42^ $\:{h}_{3}=\frac{{k}_{ad}}{{x}_{ad}}\left(1-\frac{{D}_{o}}{w}\right)$ $\:{h}_{4}=\frac{{x}_{c}}{\frac{{w}^{2}}{8{k}_{c}}+\frac{{x}_{ad}}{{k}_{ad}}\frac{{x}_{c}w}{{D}_{o}}}$ $\:{h}_{5}=\frac{8{k}_{abs}}{w-{D}_{0}}\frac{{x}_{abs}}{w}$ $\:{h}_{6}=\frac{2{k}_{ins}}{{x}_{ins}}\left(1-\frac{{D}_{o}}{w}\right)$ $\:{h}_{7}=\frac{1}{\frac{w}{{h}_{9}\pi\:{D}_{i}}+\frac{w}{{{C}^{*}}_{bo}}}$ $\:{h}_{8}=\frac{2{k}_{ins}}{{x}_{ins}}\left(\frac{\pi\:}{2}+1\right)\frac{{D}_{o}}{w}$ $\:{h}_{9}=4.36\frac{{k}_{f}}{{D}_{h}},\hspace{0.25em}\hspace{0.25em}\text{\:for\:}{Re}^{t}<2300$ (laminar) ^33^ $\:{{h}_{10}}^{t}=\frac{1}{\frac{{x}_{ins}}{2{k}_{ins}}+\frac{1}{{{h}_{1}}^{t}}}$	$\:\left(\alpha\:\tau\:\right)\cong\:1.01\left({\alpha\:}_{c}{\tau\:}_{g}\right)$ ^40^ $\:{{T}_{s}}^{t}=0.0552{{{T}_{a}}^{t}}^{1.5}$ ^43^ $\:{{\eta\:}_{ele}}^{t}={\eta\:}_{STC}\left[1-{\beta\:}_{c}\left({{T}_{c}}^{t}-{T}_{c,ref}\right)\right]$ ^44^ $\:{D}_{h}={D}_{i}$ $\:{D}_{i}={D}_{o}-2{x}_{t}$ $\:{{C}^{*}}_{bo}=\frac{\left({k}_{bo}{w}_{bo}\right)}{{x}_{bo}}$ ^45^ $\:\left.{{T}_{f}}^{t}=0.5{\left(T\right.}_{co-in}+{{T}_{co-out}}^{t}\right)$

For the assumed laminar flow, the Reynold’s number, $\:{R}_{e}$ is calculated according to:

22 $$\:{Re}^{t}=\frac{{\rho\:}_{f}{{v}_{f}}^{t}{D}_{i}}{{\mu\:}_{f}}$$

$\:{v}_{f}$ is the average fluid velocity (m/s) calculated as:

23 $$\:{{v}_{f}}^{t}=\frac{0.001{\dot{m}}^{t}}{\left(\frac{{{\pi\:D}_{i}}^{2}}{4}\right)}$$

From Fig. 3a, it should be noted that there are two pathways via which heat flows from the PV to the tube-bond position: first, through the adhesive layer, and then horizontally along the absorber plate, $\:{({Q}_{4}}^{t}$ and $\:{{Q}_{7}}^{t})$ and furthermore, via the adhesive layer, then vertically through the absorber at the tube-bond location, $\:\left({{Q}_{5}}^{t}\right)$. In the thermal model, Eq. (8) accounts for the minimum temperature difference between the hot and cold steam, (9) is for the mean working fluid temperature, (10) accounts for the heat gained by the fluid, while (11) relates the cell temperature to the mass flow rate; Eq. (12) considers the net heat flux at the PV layer $\:{({Q}_{net}}^{t})$, the incident heat flux on the PV/T surface $\:{({Q}_{1}}^{t})$, convection loss to the ambient $\:{({Q}_{2}}^{t})$, radiation loss to the sky $\:{({Q}_{3}}^{t})$, conduction to the absorber $\:{({Q}_{4}}^{t})$, and tube $\:{({Q}_{5}}^{t})$, and energy flux converted to electricity $\:{({Q}_{6}}^{t})$; Eq. (13) considers PV to absorber $\:{({Q}_{4}}^{t})$, absorber to the outer tube surface $\:{({Q}_{7}}^{t})$, and absorber to insulation $\:{({Q}_{8}}^{t})$ interaction by conduction; Eq. (14) considers conduction from the PV to the tube $\:{({Q}_{5}}^{t})$, absorber to the outer tube surface $\:{({Q}_{7}}^{t})$, tube to insulation $\:{({Q}_{10}}^{t})$, and convection from the tube to the working fluid $\:{({Q}_{9}}^{t})$; Eq. (15) caters for heat transfer between the absorber $\:{({Q}_{8}}^{t})$, insulation $\:{({Q}_{10}}^{t})$, and loss to the ambient $\:{({Q}_{11}}^{t})$; in (16), the heat gained by water $\:{({Q}_{12}}^{t})$ is equal to that lost by the tube $\:{({Q}_{9}}^{t})$ by convection. The thermo-physical properties of water are temperature-dependent and are given in Table 9.

**Table 9:** Water property relation used for temperatures between 290 K and 370 K^4^.

Property	Relation	Unit
Thermal conductivity	$\:{k}_{f}=-0.000007843{T}_{co-in}^{2}+0.0062{T}_{co-in}-0.54$	W/mK
Specific heat capacity	$\:{c}_{f}=-0.0000463{T}_{co-in}^{3}+0.0552{T}_{co-in}^{2}-20.86{T}_{co-in}+6719.637$	J/kgK
Density	$\:{\rho\:}_{f}=-0.003{T}_{co-in}^{2}+1.505{T}_{co-in}+816.781$	kg/m^3^
Dynamic viscosity	$\:{\mu\:}_{f}=1.788\times\:{10}^{-3}exp\left(-1.704-\frac{1448.538}{{T}_{co-in}}+\frac{521926.58}{{T}_{co-in}^{2}}\right)$	kg/ms

Additionally, (17) considers a fully mixed, non-thermal stratified storage with no water drawn with energy fluxes shown in Fig. 3b. Hence, the heat supplied by the PV/T $\:{({Q}_{13}}^{t})$, storage heat losses to the ambient $\:{({Q}_{14}}^{t})$, and consumption $\:{({Q}_{15}}^{t})$ are accounted for. In (18), at all times the layer temperatures are higher than ambient temperature; (19) eliminates counter-flow in PV/T operation; (20) gives a threshold to the fluid inlet temperature, while (21) accounts for laminar flow as assumed. The superscript (^t^) denotes time dependence in hours.

#### PV/T system performance analysis

For performance analysis of the PV/T system, its efficiency is computed in relation to the insolation on its top plane.

24 $${\text{Electrical performance}}:\:{{\eta\:}_{ele}}^{t}=\frac{{{Q}_{6}}^{t}}{\left(wL\right){G}^{t}}$$

25 $${\text{Thermal performance}}:\:{{\eta\:}_{th}}^{t}=\frac{{{Q}_{12}}^{t}}{\left(wL\right){G}^{t}}$$

#### Solving approach

**Fig. 4:**
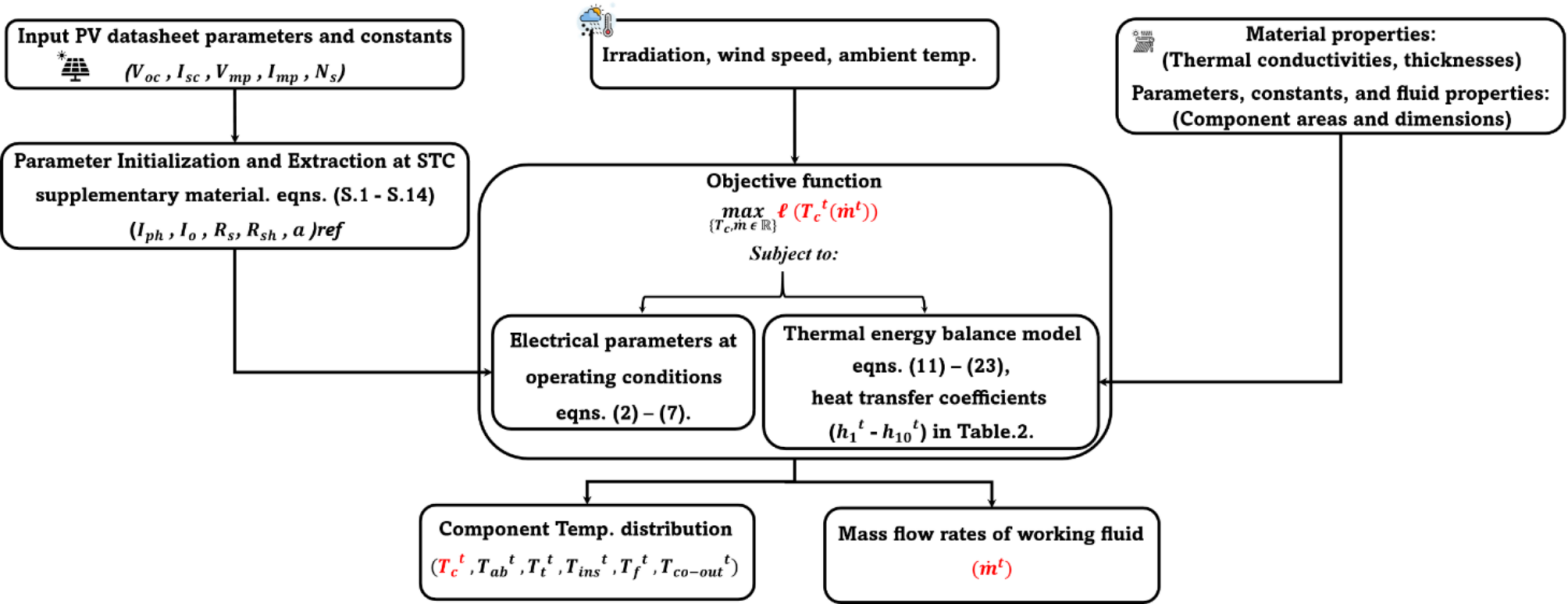
Thermal model and modified electrical parameter flow diagram.

A diagram of the numerical process is shown in Fig. 4. Firstly, the electrical, thermal, material properties, constants, and meteorological data are input. The electrical model is initiated to determine the parameters at STC. Then, the temperature of each layer is determined from the thermal model. Finally, the parameters are determined at operating conditions for the corresponding optimal power output.

**Fig. 5:**
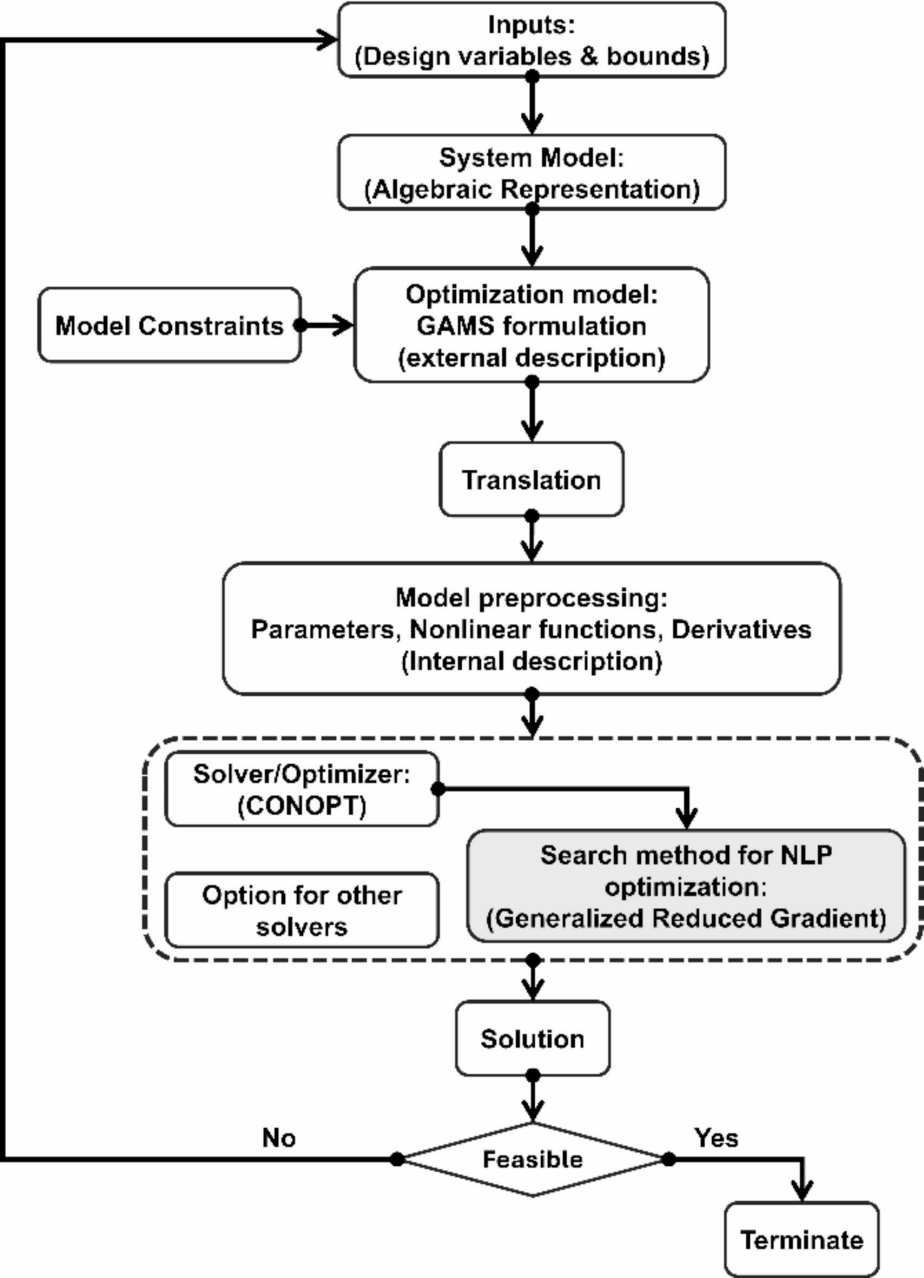
GAMS Nonlinear problem optimization scheme.

The optimization problem in Eq. (1) presents nonlinearities both in the objective function described by the exponential term (a nonlinear function) and nonlinear constraints. Therefore, CONOPT^46^, a built-in General Algebraic Modelling System (GAMS) solver specifically designed for Nonlinear programming (NLP), is used to solve the problem. The optimization scheme for the presented problem is shown in Fig. 5.

**Fig. 6:**
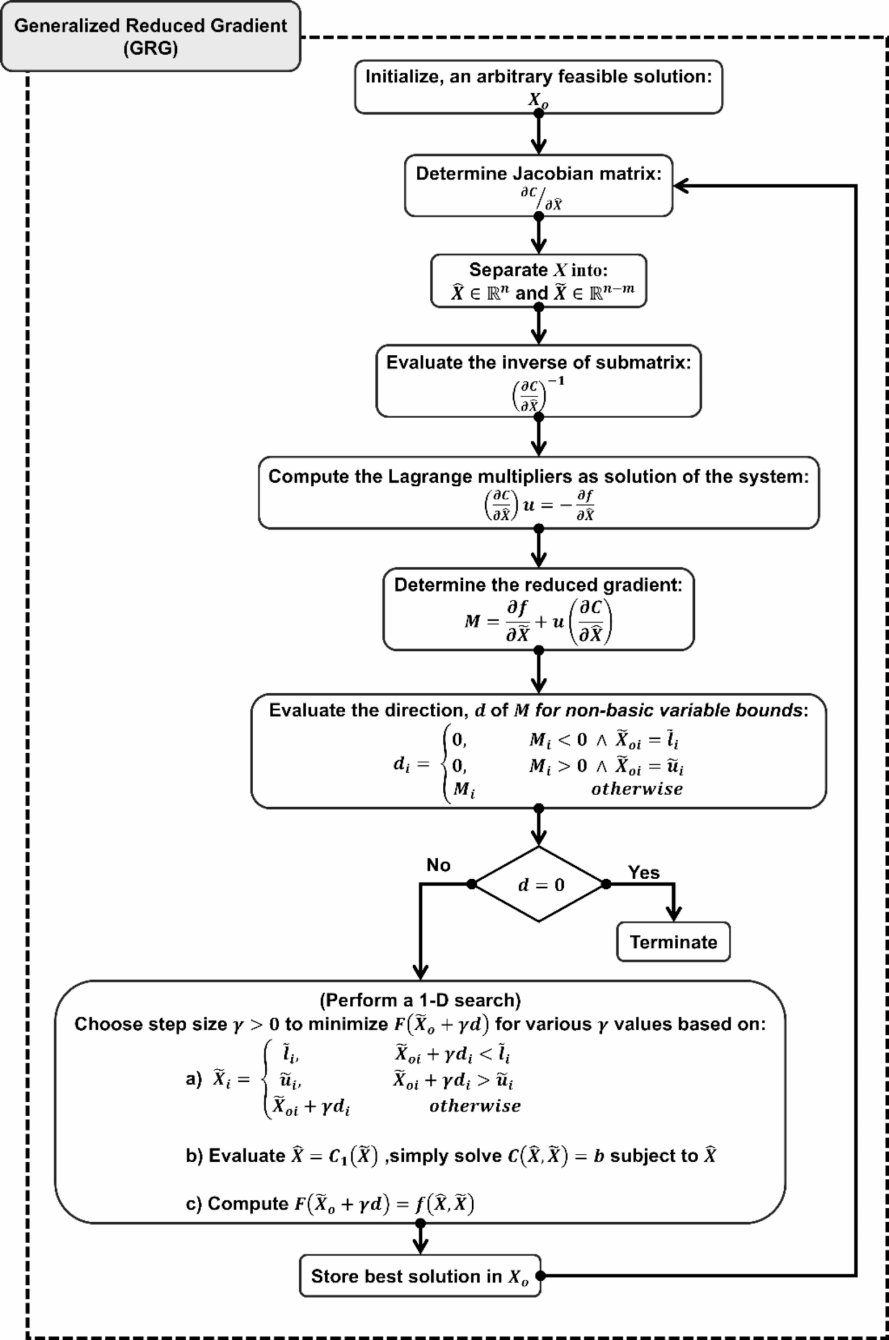
Generalized reduced gradient algorithm^49^.

In Fig. 5, CONOPT is based on the Generalized Reduced Gradient (GRG) algorithm^47^, which iterates through the feasible region seeking to find the optimal solution to the objective function. Moreover, it has other algorithms out of which it can automatically select based on the problem, if not specified^48^. The details of the GRG algorithm are presented in the supplementary material S.2, and its flowchart is summarized in Fig. 6.

### Dynamic power simulation of hybrid microgrid including PV/T

**Fig. 7:**
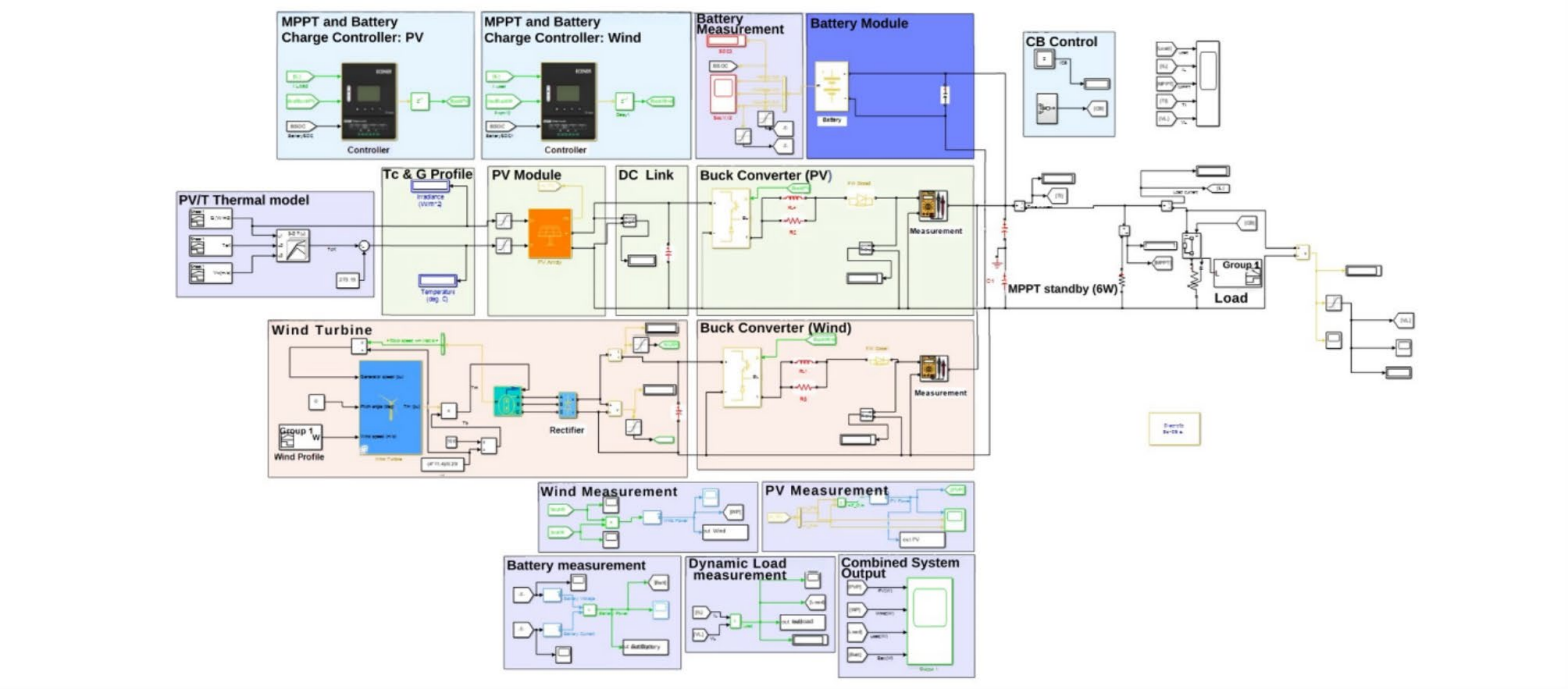
Microgrid model in Simulink.

The microgrid was implemented in MATLAB/Simulink, as depicted in Fig. 7.

The constituent components are as follows:

#### PV/T module

The PV/T module (250 W), whose electrical characteristics are shown in Table 2. However, since the PV/T component is not available in the Simulink library, it can either be made with MATLAB functions or component interconnection. Using the determined electrical parameters and mass flowrate with some material properties obtained from similar works as presented in Tables 3 and 4, the sheet & tube PV/T user-defined component mask was developed, as shown in the supplementary material S.3 (Figure S.1.(a)). The inputs are irradiation-G (W/m^2^), wind velocity-V*w* (m/s), and ambient temperature-Ta (°C), while the outputs include voltage (V), current (A), cell temperature (K), electrical and thermal efficiency. Computation time was a major drawback when integrated into the simulation model due to the concurrent calling of an extrinsic function for each time step. Alternatively, the PV/T system was realized with a 3-D lookup table depicted in the supplementary material S.3 (Figure S.1.(b)) and eventually evaluated with the other microgrid components.

#### Wind turbine

The wind turbine (100 W) has a 9.3 m/s base wind speed. The intakes to the Simulink component are the per unit rotor speed, pitch angle (deg.), and wind velocity (m/s). The per unit output torque is calculated using the base torque’s gain, which is based on the generator’s specifications. A four-pole PMSG, whose resultants are the rotor speed in *(rad/s)* and ac voltage, is then fed the actual torque. Housing a stator phase resistance of 25 mΩ and an armature inductance of 0.735 mH, its power terminals are then interfaced to a rectifier.

#### Battery

The storage is a 30 Ah AGM deep cycle battery with a nominal discharge current of 6 A, an internal resistance of 0.004 Ω, and a 14.4*V* full-charge voltage. A variable resistor is used to dynamically represent the load. It is interfaced to the complete model by two 66 mF capacitors to attenuate the voltage fluctuations brought on during system operation.

Along with the actual dynamic load, a 6 W load (24 Ω resistor) is paralleled to consider the MPPT controller’s idle operation. A 500 F DC link capacitor, which aims to stabilize the DC voltage, connects the PV/T module and wind power generation system to the buck converter on either side. The buck converter is made up of IGBT and diode pairs that are governed by firing pulses processed by a PWM generator. The MPPT is based on the perturb and observe (P&O) algorithm in^15^ to create the control approach.

The detailed specifications of the components are presented in Tables 5 and  6.

## Results and discussion

### PV/T optimal design model validation

**Fig. 8:**
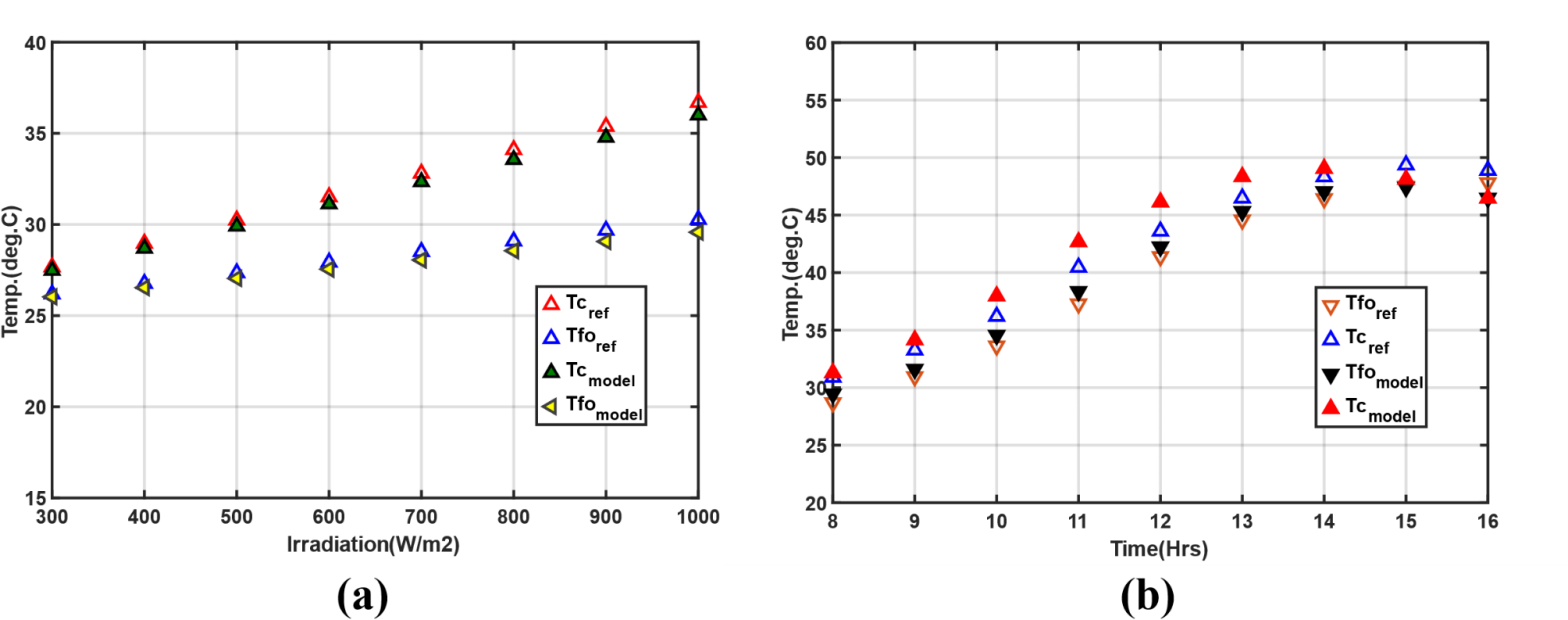
Validation of the PV/T optimal model results in; (**a**) for the unglazed PV/T with laminar flow^14^, (**b**) with Huang et al.^50^ results.

The cell temperature and fluid outlet temperature of the PV/T system estimated by the model developed in this research were compared with the simulation and experimental findings reported in^14^ and ^50^, respectively, under the same operational conditions, as seen in Fig. 8a and b. The PV/T system design configuration with specifications is given in Tables 2 and 3, and 4. Since the modeled PV/T system was unglazed, the simulation results for an unglazed PV/T in laminar flow^14^, with a constant fluid inlet temperature of 298.15 K equal to the ambient temperature, operating under a constant wind speed of 1.5*m/s* and variable solar irradiation from 300 to 1000 W/m^2^ with a fixed mass flowrate of 0.005 kg/s/tube were used. Figure 8a shows the first validation results with RMSE values for cell and fluid outlet temperatures estimated at 0.46. In^50^, the operational conditions included solar irradiation, ambient temperature, inlet water temperature, constant wind speed of 1 m/s, and fixed total mass flow rate of 0.05 kg/s. Comparing the cell and outlet water temperatures in Fig. 8b, there is tolerable congruence between the results of the current study and the reference study.

### Optimal flow rate of the working fluid

In PV/T operation, the fluid flow rate is a pivotal external factor, as it significantly contributes to the system’s performance by affecting efficiency. Due to flow conditions that are close to stagnation, the collector’s ability to transfer heat is compromised, which lowers the convective heat exchange coefficient amidst the working fluid and tubes^51^. Contrarily, even though the heat transfer process is improved with high flow, a penalty for the higher cost of pumping exists. Therefore, a tradeoff is required for optimal PV/T operation.

**Fig. 9:**
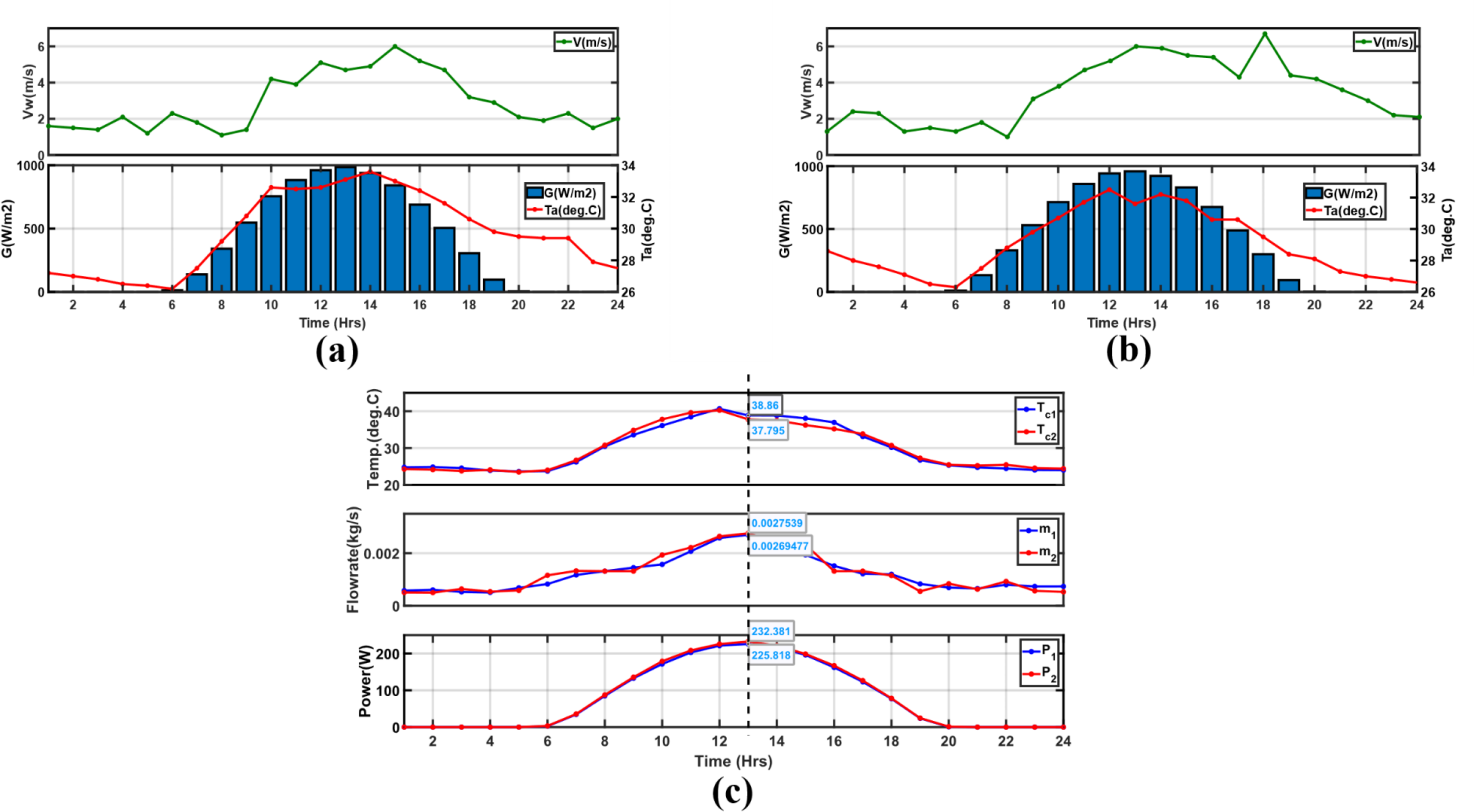
Meteorological inputs used on; (**a**) July 14th, (**b**) July 27th, (**c**) Optimal flow rate based on maximum power.

In this study, the optimal flow rate was estimated based on the given ambient conditions of the selected days of July 14th and July 27th that had received the most significant solar irradiation in Fukuoka city, Japan, in 2021, shown in Fig. 9a and b. Therefore, using the PV/T specifications in Tables 2 and 3, and 4, a tilt angle of 30°, a fluid inlet temperature of 298.15 K, and a temperature of water in the tank at 295.15 K, the flow rate that corresponds to the maximum power obtained with the explained numerical procedure was obtained. As expected, the system performs only in periods with solar irradiation between 7 h and 19 h. The three factors show an increasing trend, peaking at around 13 h before decaying. Flowrate (m_2_) was highest at 13 h due to maximum irradiation that reduced the water density, increasing buoyant system pressure and reducing cell temperature. As a result, the corresponding power on July 27th (*P*_*1*_) was also at its maximum at around 232 W compared to 225 W on July 14th (*P*_*2*_). With this, the flow rate (m_2_) of 0.0028 kg/s per tube (0.028 kg/s total flow rate) corresponding to 232 W and 37.8 °C at 13 h was taken as the optimal in this research Fig. 9c, as summarised in Table 10. The study doesn’t consider pressure drop analysis related to pumping requirements; hence, the obtained flow rate is compared with those from studies specified in Table 11. Moreover, a 1 °C temperature change resulted in a 6.56 W corresponding power increment at 13 h between the two days.

**Table 10:** Variation of the objective function with flow rate at 13 h.

Day	Flowrate (kg/s/tube)	Tc (°C)	Power (W)
July 14th	0.002695	38.860	225.818
July 27th	0.002753	37.795	232.381

**Table 11:** Flow rate comparison with other studies (excluding the effect of pressure drop).

Technology	Flow rate (kg/s)	Ref.
PV/T (sheet and tube)	0.029	^33^
PV/T (sheet and tube)	0.025	^45^
PV/T (serpentine)	0.020	^52^
PV/T (sheet and tube)	0.01–0.02	^53^
PV/T (sheet and tube)	0.06	^54^
PV/T (sheet and tube)	0.028	This study

### PV/T technical performance analysis compared with a typical PV system

**Fig. 10:**
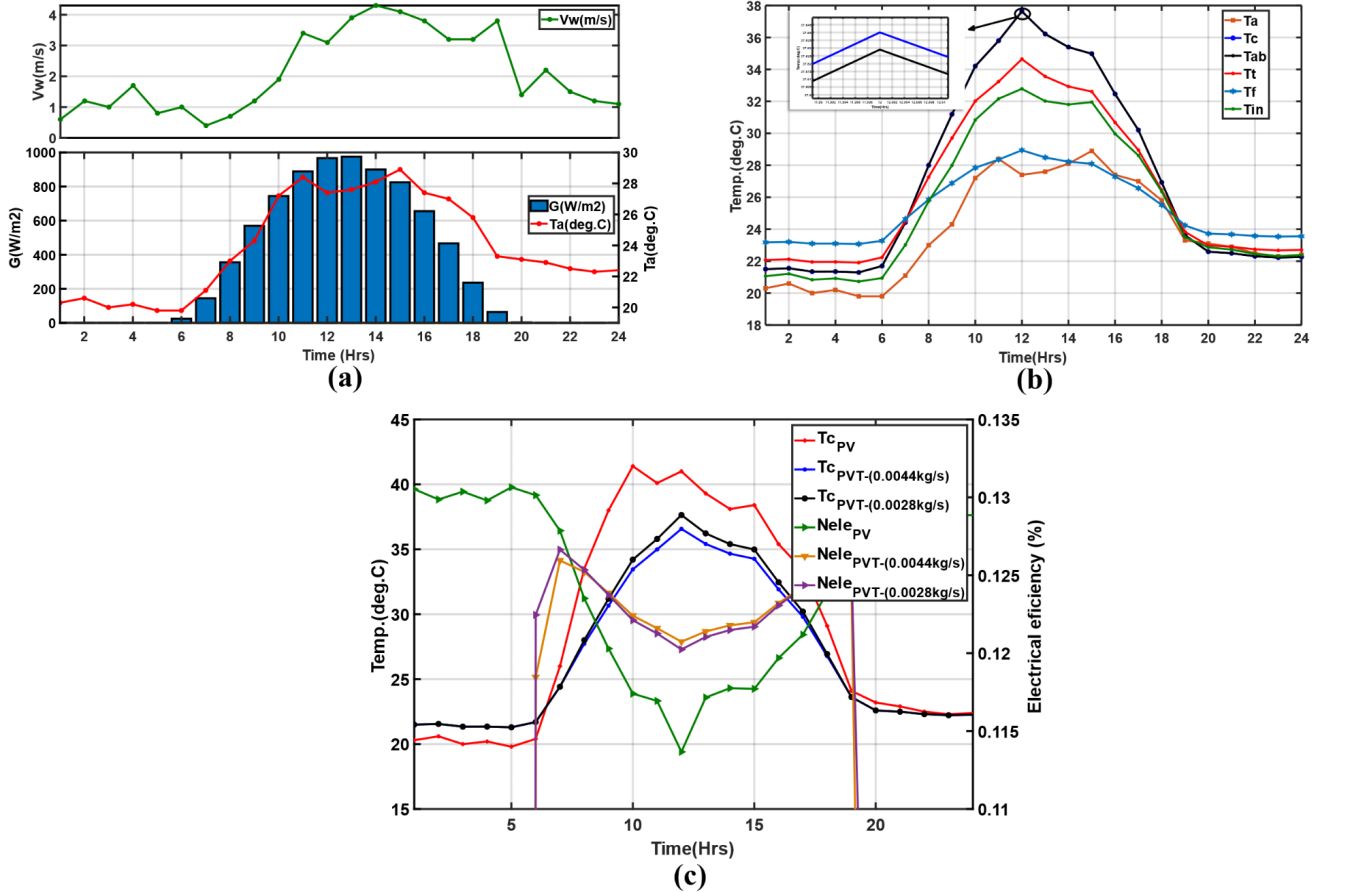
(**a**) Meteorological data, (**b**) Layer temperature distribution, (**c**) $\:{\eta\:}_{ele}$vs. $\:{T}_{c}$ for PV and PV/T at specified flow rates.

Here, the PV/T and PV systems were compared in terms of cell temperature and electrical efficiency, using meteorological data shown in Fig. 10a, considering the estimated optimal flow rate (0.0028 kg/s per tube) and inlet water temperature of 298.15 K. The component temperature distribution (cells, absorber, tube, fluid, insulation, and ambient) is depicted in Fig. 10b. As solar irradiation increases, the temperatures also rise but are distributed based on material properties such as conductivity and thickness in the module, peaking at 12 h with about 966 W/m^2^. The cell layer and the absorber are 37.64℃ and 37.63 °C respectively, higher than the other components. The module cools, and the temperatures drop at sunset, when the solar irradiation and the surrounding temperature drop. The electrical efficiency of the PV cell is significantly reliant on operating temperature, which is influenced by fluid temperature. Figure 10c conveys the variation of electrical efficacy and operating temperature for a PV and PV/T at two flow rates. The optimal flow rate in this research and the one reported in^9^ (0.0044kg/s per tube) is a reference value in EN12975, which is the standard for collector mass flow rate. It is evident that at midday, the temperature of the $\:PV$, $\:{PV/T}_{0.0044}$ and $\:{PV/T}_{0.0028}$ reaches 41 °C, 36.57 °C and 37.64 °C respectively, resulting in efficiencies of 11.36%, 12.07%, and 12.02%, respectively with temperature reductions summarized in Table 12. With this, it is evident that the PV/T outperforms a conventional PV considerably due to the cooling effect of the cells enhancing increased efficiency.

**Table 12:** Summary of maximum temperature reduction comparison between $\:PV$, $\:{PV/T}_{0.0044}$ and $\:{PV/T}_{0.0028}$

$\:{\varvec{T}}_{\varvec{P}\varvec{V}}$	Flowrate/tube (kg/s)	$\:{\varvec{T}}_{\varvec{P}\varvec{V}/\varvec{T}}$	Temperature (%)*
41	0.0044	36.57	− 11.7
41	0.0028	37.64	− 9.1

*(–)Indicates a temperature reduction.

### PV/T performance evaluation in a hybrid solar/wind/battery microgrid

**Fig. 11:**
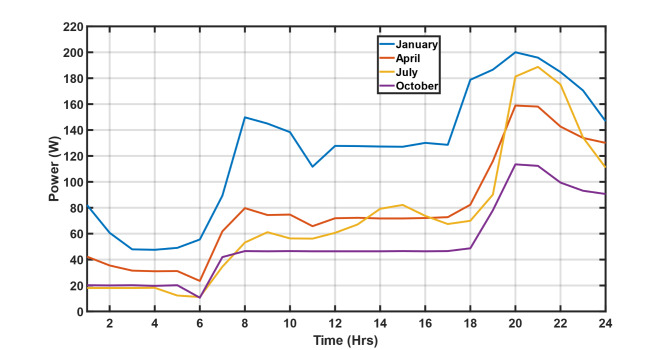
Defined seasonal-daily load profiles.

In this subsection, the PV/T system represented by a 3-D lookup table is integrated, forming a PV/T/Wind/Battery microgrid and simulated for a period of 72 h based on four scenarios and compared with the conventional PV/Wind/Battery microgrid. The short period was considered to capture electrical events such as system oscillations and transients. Moreover, three days were taken as adequate to check whether the system can support its load given a day of autonomy. Additionally, the 72-hour period was chosen based on an extensive analysis of 2021 annual weather data from Fukuoka-Japan^55^, as seen in the supplementary material S.4. In Fig. 11, the daily load profiles are dynamically defined to depict demand in four annual seasons. Therefore, the representative months correspond to typical seasons with extreme weather events that dictate consumer consumption patterns, such as January (Winter), April (Spring), July (Summer), and October (Autumn).

**Fig. 12:**
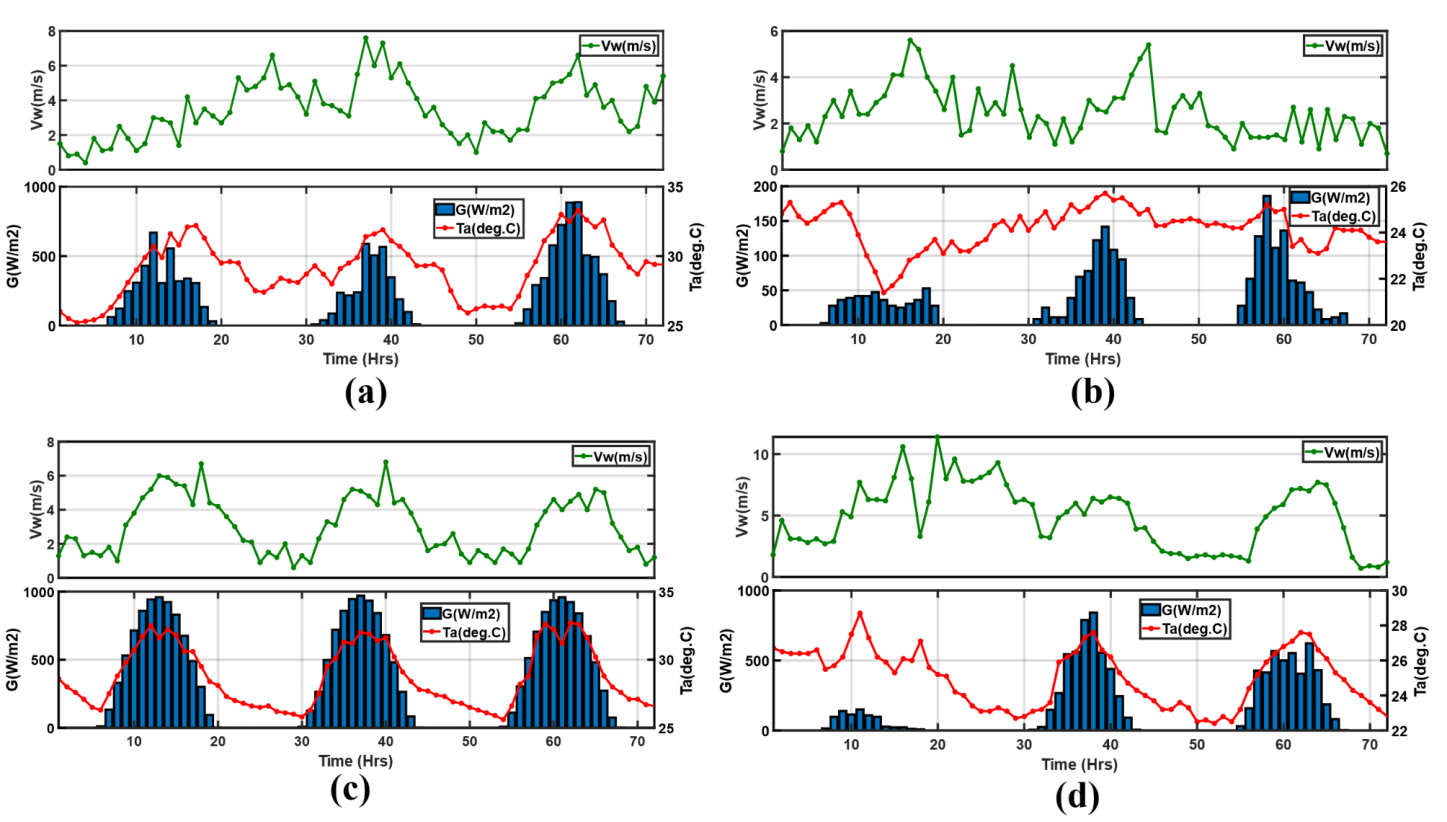
Meteorological conditions for; (**a**) cloudy, (**b**) rainy, (**c**) sunny, and (**d**) windy periods.

The meteorological conditions that define the operation scenarios (cloudy, sunny, rainy, and windy) used in the performance analysis of the microgrid are depicted in Fig. 12a–d. In support of the scenario classification, a parallel exhaustive data analysis was done. As such, the sunny period had the highest irradiation levels, whereas the windy period had the greatest mean wind speeds. The cloudy period had reduced irradiation due to cloud cover, and the rainy period had the least irradiation due to weather conditions. Intuitively, all periods were selected so that at any one time, there is either solar or wind energy production.

#### Cloudy period

**Fig. 13:**
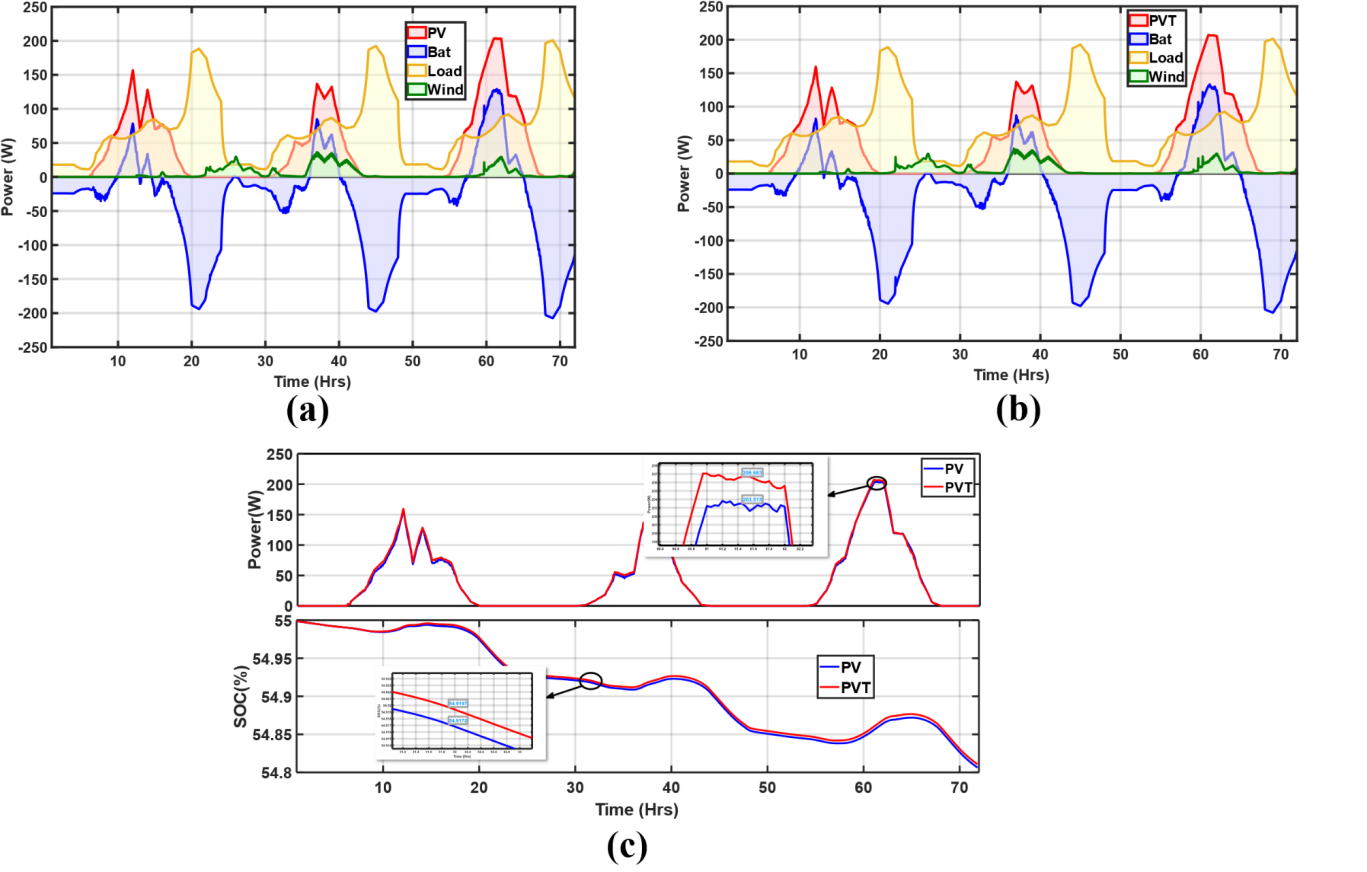
Energy balance of the microgrid with (**a**) PV and (**b**) PV/T; (**c**) PV and PV/T comparison with respect to generation and SoC (Cloudy).

The energy balances are distinctly presented in Fig. 13a and b for the PV and PV/T. Both show similar profiles. Generally, the load starts peaking from dusk at around 20 h to about 190 W at 21 h for the first day. The electricity generation is most significant during the day, when there is adequate solar irradiation with moderate wind speeds, and the peak generation from PV/T and PV is about 207 W and 203 W, respectively, on the third day. However, the wind generation peaks at 36 W for both cases but is on the second day. As expected, in periods of insufficient generation, supply comes from the battery. In Fig. 13c the PV/T and PV are comprehensively compared for all periods. It turns out that the PV/T outperforms the typical PV system due to the cooling of the cells enhancing power output. On day 3 at noon, the PV/T generated about 1.6% more power than the PV. Regarding the SoC battery, the PV/T performed the best since the battery was not discharged as much as the PV microgrid. Additionally, the battery’s SoC is reduced because of the evening peak load, which considerably depletes the battery.

#### Rainy period

**Fig. 14:**
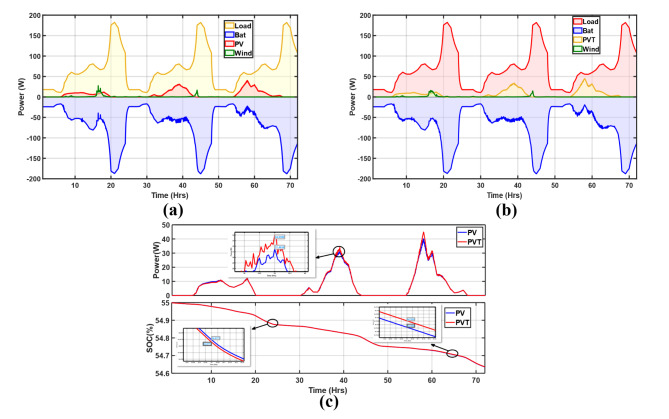
Energy balance of the microgrid with (**a**) PV and (**b**) PV/T; (**c**) PV and PV/T comparison with respect to generation and SoC (Rainy).

The irradiation in this scenario was the least, reaching a maximum of just over 186 W/m^2^. The slightest wind energy generation also characterized this scenario due to the low mean wind velocity of nearly 2.4 m/s. Figure 14a and b show the PV and PV/T energy balance. Typically, the maximum generation from PV was about 39.5 W and 44.9 W for the PV/T, both on day 3, while the last day barely had any wind power generation. In this period, the battery mainly operated as a main and backup power source throughout the 3 days. Regardless of the lowest total generation, as shown in Fig. 14c, the PV/T still generated more power than the PV system, with about 2.6 W more at 39 h on day 2. However, on day 1, it was nearly the same since the irradiation was minimal at about 52 W/m^2^. Since, in this scenario, there was prolonged battery operation, its SoC had the worst profile with the lowest charging rates, and this is not a recommended battery system operation as it contributes to cycle loss due to deep discharge.

#### Sunny period

**Fig. 15:**
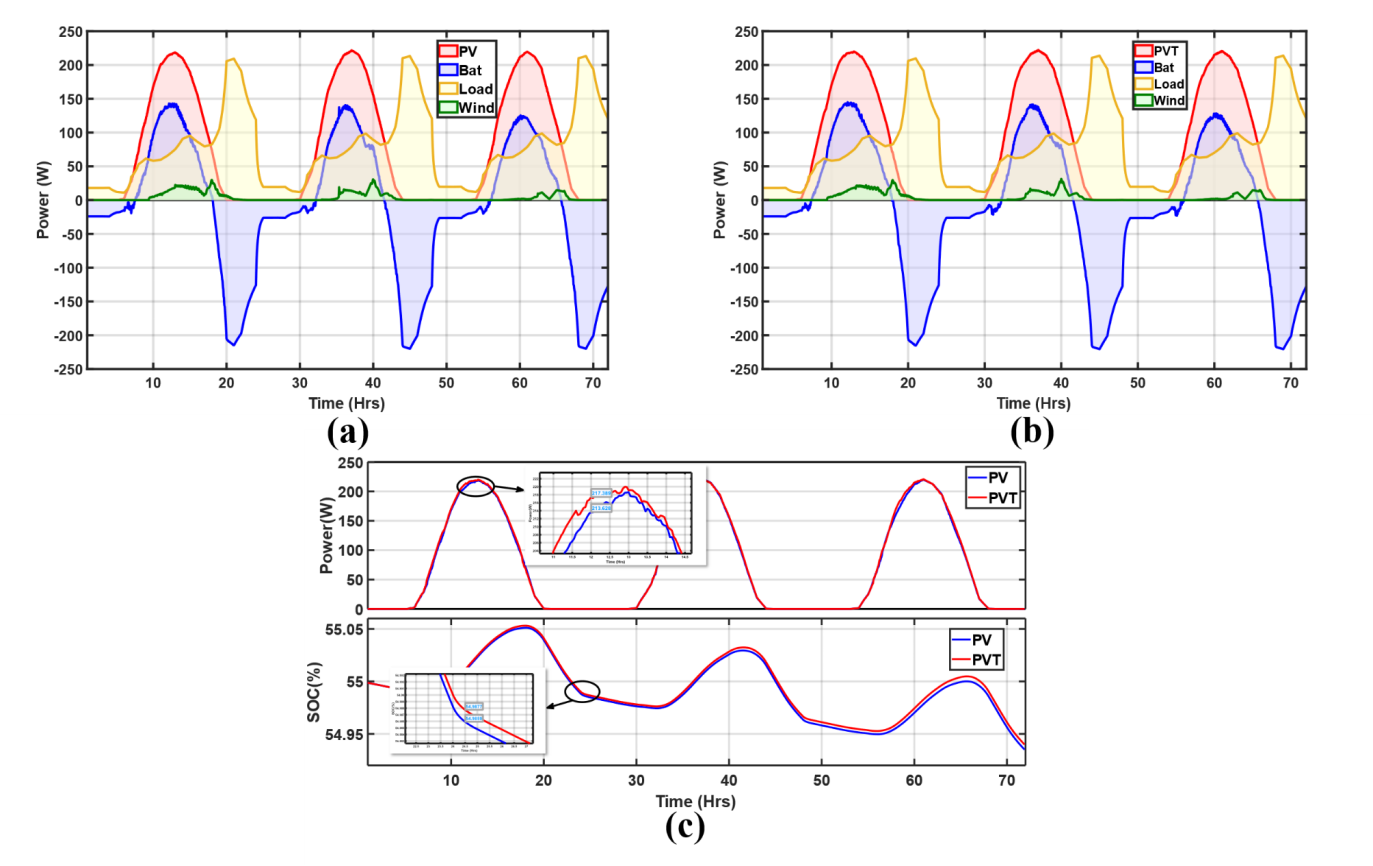
Energy balance of the microgrid with (**a**) PV and (**b**) PV/T; (**c**) PV and PV/T comparison with respect to generation and SoC (Sunny).

Here, the irradiation is most significant on all days compared to the remaining scenarios, with wind speed reaching peaks of 6.8 m/s on day 2. As shown by the energy balances in Fig. 15a and b the battery is only supplied when there is no generation from solar and wind, especially during the evening hours. During the day, the generation is highest at about 221.24 W for the PV and 222.26 W for the PV/T, all on day 2. The load is fully met in addition to battery charging, as shown by the SoC profiles in Fig. 15c. Wind generation is also present but is insignificant compared to solar generation; it is at its maximum at about 30.49 W on day 2. Figure 15c shows the PV and PV/T power generation and SoC comparison; in all 3 days, the PV/T generated more power; about 1.76% more power is generated at midday on day 1. The SoC is significantly maintained, regardless of the declining trend due to the evening load. Hence, there is a projection of improved battery life due to no discharging during the day, which maintains high SoC levels.

#### Windy period

**Fig. 16:**
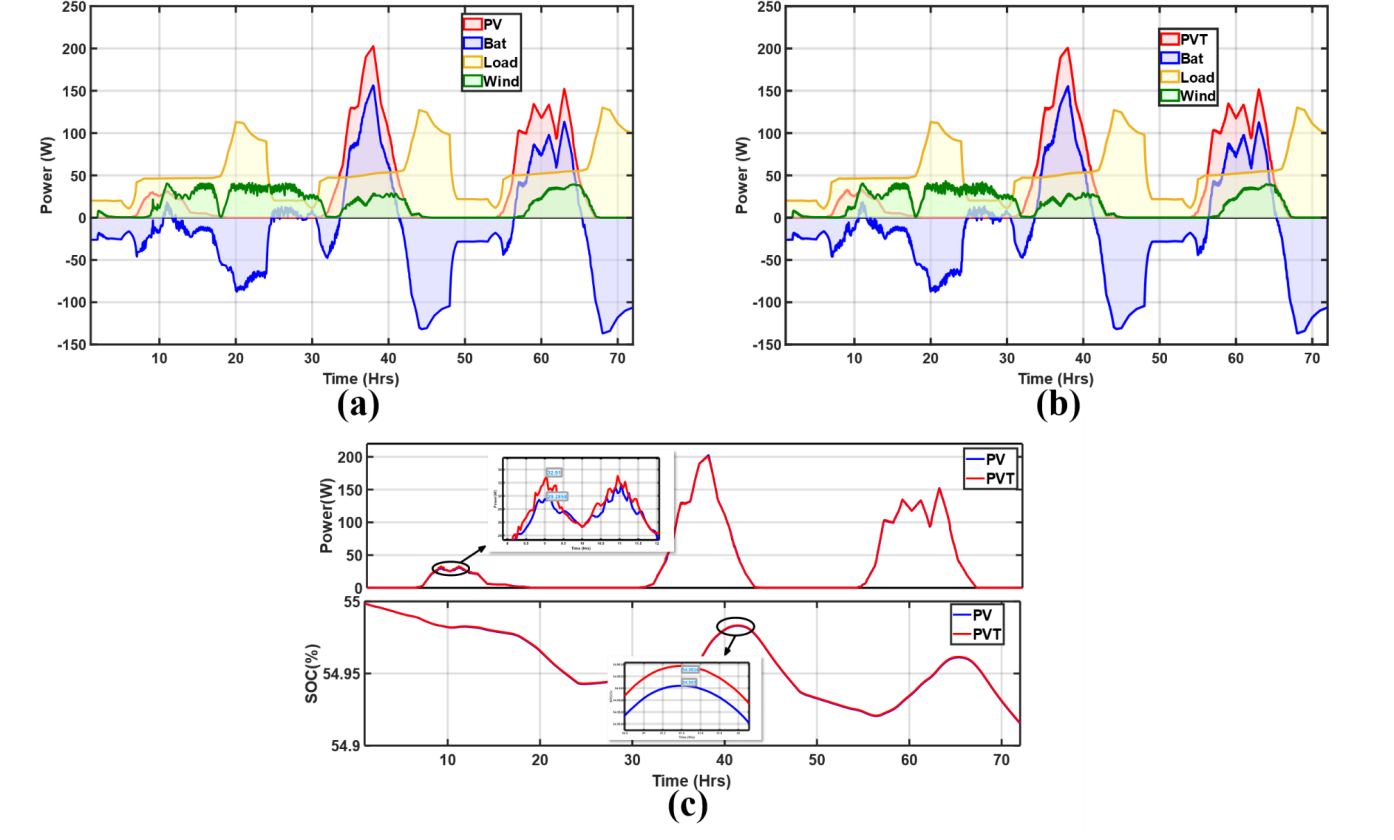
Energy balance of the microgrid with; (**a**) PV and (**b**) PV/T; (**c**) PV and PV/T comparison with respect to generation and SoC (Windy).

This period corresponded to autumn and, therefore, had a different load profile from the 3 scenarios in summer. As depicted in Fig. 16a and b wind power generation is prevalent on day 1 with a slight dip at 18 h to a wind speed of about 3 m/s, resulting in a steady decline in power from nearly 37 W at 15 h to about 0.8 W at 16 h. This wind speed was close to the turbine cut-in speed. There was minimal solar generation on day 1; the maximum was about 32.08 W. However, on day 2, there was a significant increase in solar power generation at about 200 W at 38 h. In Fig. 16c, as expected, the PV/T still outperforms the PV in the 72 h period, just like in the other three scenarios. As seen in the zoomed section on day one, 3.62 W more is generated by the PV/T at 9 h. Still, the SoC depicts low charging on day one, while significant charging peaks are observed on days 2 and 3. This is because of the increased generation.

### Scenario electricity generation and PV/T operational reliability

**Fig. 17:**
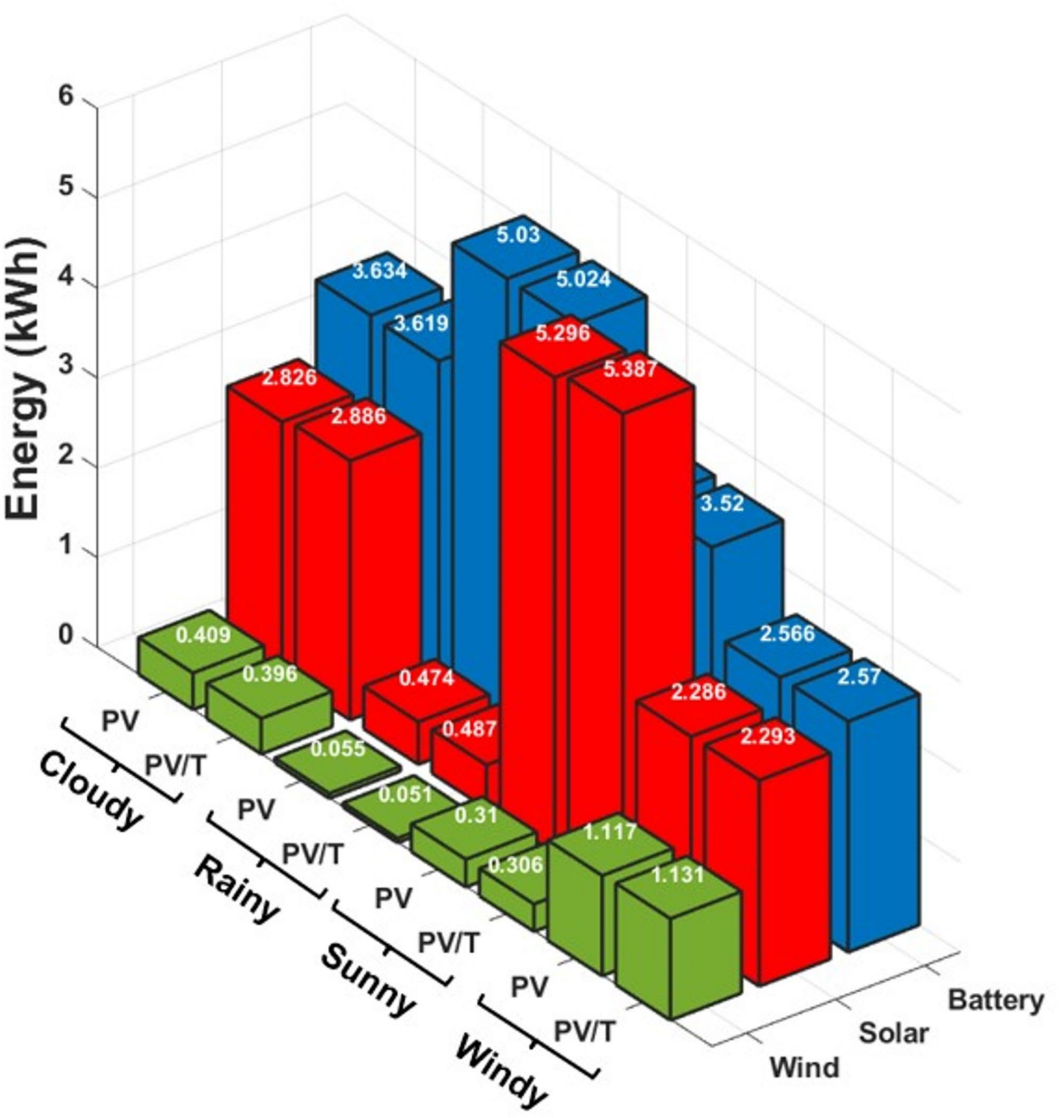
Total daily electricity production in the scenarios.

Figure 17 shows the total daily electricity production for the scenarios, highlighting the PV and PV/T systems. The plot only shows generation energy sources, including solar, wind, and battery discharge, as they act as a source. The rainy scenario has the slightest wind and solar power generation for both the PV and PV/T but, surprisingly, the highest battery power. This is because of insufficient primary generation to meet the load; hence, the battery provides the required power with the peak discharge of 5.03 kWh using the PV module. The sunny scenario has the highest solar generation contribution of 5.296 kWh and 5.387 kWh for PV and PV/T, respectively. It also has the highest total generation from wind and solar technologies, about 5.606 and 5.694 kWh from PV and PV/T, respectively. Windy and cloudy scenarios have nearly the same combined generation of about 3.282 and 3.424 kWh for PV/T. However, the variation is attributed to the difference in load profiles, as the windy scenario has a lower total combined load than the cloudy scenario. Among the sources, wind power is the least due to the small capacity of the wind turbine generator, which would have been most remarkable in the windy scenario.

Additionally, the operational reliability is assessed based on the system’s self-sufficiency ratio, which determines the microgrid’s capability to meet its demand, as reported in Table 13. It can be observed that, for the proposed PV/T-based microgrid in this study, the self-sufficiency ratio in all operational scenarios is above 1. This indicates that the proposed system can efficiently and reliably operate under various conditions while meeting user expectations.

**Table 13:** PV/T-based microgrid self-sufficiency.

Scenario	Cumulative generation (kWh)				Cumulative consumption (kWh)				Sufficiency ratio
	PV/T	Wind	Discharging	Total	Demand	MPPT	Charging	Total	
Sunny	5.39	0.31	3.52	9.22	5.89	0.48	2.48	8.85	1.04
Cloudy	2.89	0.40	3.62	6.91	5.41	0.45	0.84	6.70	1.03
Windy	2.29	1.13	2.57	5.99	4.02	0.46	1.28	5.76	1.04
Rainy	0.49	0.05	5.02	5.56	5.10	0.43	0.00	5.53	1.00

The average cell temperature reduction, along with the excess generation of the microgrid with PV/T compared to the microgrid with PV, is reported in Table 14, and hourly generation in the supplementary material (Table S.1). Generally, a reduction in cell temperature contributes to an enhancement in power production due to cooling but is also affected by its microclimate especially the heat index, insolation, and PV/T design features. The cloudy scenario has a higher extra generation than the sunny scenario because the average cell temperature reduction is about 2% more. This is attributed to the higher convection heat transfer to the ambient, $\:{{h}_{1}}^{t}$. The windy period has the least excess generation as compared to the rest. This is due to the lower cell temperature reduction regardless of the moderate operating conditions, which can be attributed to the low convection heat transfer rate; hence, the PV/T operates nearly like the conventional PV system. Interestingly, the rainy period generates the highest extra power regardless of the relatively insignificant cell temperature reduction compared to the cloudy and sunny scenarios. This scenario received the least solar irradiation for the entire 3 days, slightly influencing heat generation. Therefore, this accounts for less heat accumulation within the PV/T module, hence low cell temperature and enhanced generation.

**Table 14:** Average $\:{T}_{c}$ and extra electricity from the microgrid with PV/T compared to that with PV in 72 h.

	Cloudy	Rainy	Sunny	Windy
Average surface temperature reduction (%)	− 12.75	− 4.94	− 10.60	− 3.51
Extra electricity (%)	2.12	2.74	1.72	0.31

### System operation impact on battery life

**Fig. 18:**
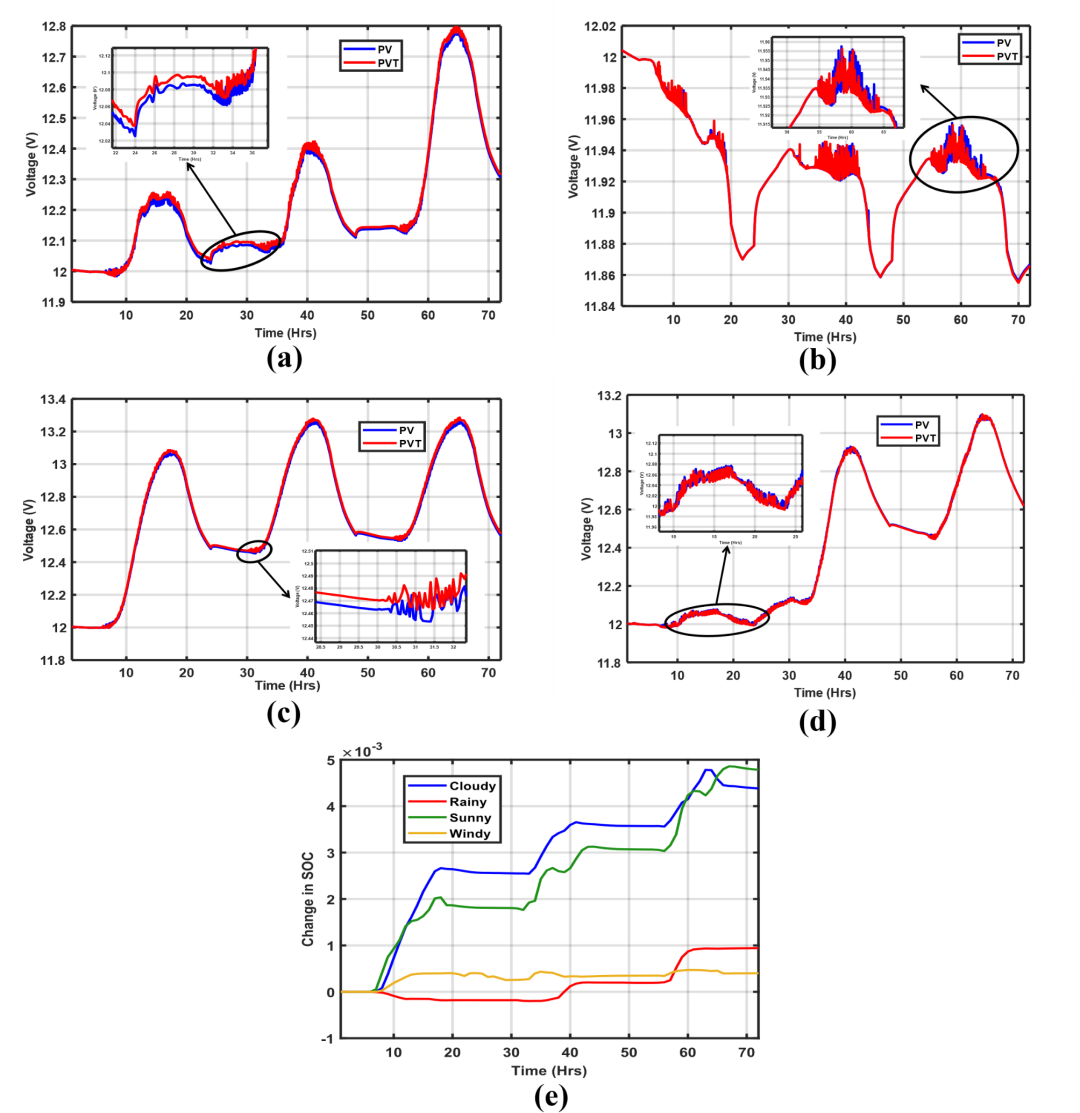
PVT vs. PV battery voltage scenario profiles: (**a**) Cloudy, (**b**) Rainy, (**c**) Sunny, (**d**) Windy, and (**e**) Change in SoC between the PV/T and PV-based system battery.

As highlighted in^24^, battery life is related to how much a battery is consumed (DoD). Therefore, increasing DoD reduces battery life. In Fig. 18a–d, battery operation profiles for the defined simulation periods are depicted. Generally, the PV/T-based microgrid battery shows improved voltages fluctuating between 12 and 13.3 V in all cases due to the increased energy yield. This is the exception of the rainy scenario in Fig. 18b, where the battery was discharged to nearly 11.8 V for both PV and PV/T-based configurations. It can also be observed that the voltage ripples are more significant, indicating an undesirable contribution to system operation. This can be attributed to the increasing stochastic load that necessitates high discharge currents, yet insufficient generation to support the battery, hence increased deep discharge. Figure 18e also affirms this by the negative change in SoC between 10 h and 40 h, which signifies poor operation.

However, the battery performance can either be maintained or enhanced by using it efficiently. This is done by avoiding high DoD to extend the battery’s end-of-life. Therefore, with the use of PV/T, as depicted in Fig. 18e, for the cloudy, sunny, and windy scenarios, the positive change in SoC indicates a significant contribution to battery life bounded at about 0.005%, equivalent to 1.8 Wh for the considered simulation duration. Another option is to adopt advanced energy storage systems such as supercapacitors, which can effectively absorb sudden spikes in energy demand (as illustrated in Fig. 18b and d). Supercapacitors can charge and discharge significant amounts of energy quickly. This ability reduces stress on the battery, thereby enhancing both battery performance and the overall operation of the microgrid^23^.

### PV/T system economic assessment

The PV/T system’s virtues make it essential to evaluate and estimate the economic benefits to make well-informed investment decisions. Considering a project whose life span is *N* years, the Levelized Cost of Energy (LCOE) is determined using^51,56,57^:

26 $$\:LCOE=\frac{{C}_{\text{fixed\:}}+\sum\:_{n=1}^{N}\:\frac{{C}_{op-n}}{{\left(1+{r}_{d}\right)}^{H}}}{\sum\:_{n=1}^{N}\:\frac{{E}_{tot-n}}{{\left(1+{r}_{d}\right)}^{n}}}$$

The economic assessment of the proposed PV/T system is based on the cost analysis presented in Table 15. The LCOE, accounting for both annual electrical energy generation of 242.62 kWh and annual thermal energy utilization of 830.31 *kWh*, is estimated at 0.26 $/kWh (40.51 yen/kWh), which is higher than the current electricity tariff in Japan (25 yen/kWh).

**Table 15:** Cost analysis of the proposed PV/T-based microgrid.

Parameter	Details	Value	Unit	Source
PV/T system (250 W)				
Site improvement, C_i_		25	$/m^2^	^57^
PV/T		392	$/m^2^	^58^
PV/T cell area		1.6	m^2^	^59^
PV/T absorber area		1.2	m^2^	^59^
Storage Tank with heat exchanger	100 litres	7	$/liter	^59^
Heat transfer fluid, C_h_		60	$/m^2^	^57^
Panel rack installation		10	% of PV/T cost	^58^
Power controller		50	$	^58^
Cabling and fixtures		67	$	^59^
Closed loop set and piping		109	$	^59^
Full installation and commissioning		197	$	^59^
Operation and maintenance	Per annum	5	% of in-service PV/T total cost	^58^
Wind turbine system (100 W)				
Capital cost		8,665	$/kW	^60^
System installation		5	% of total capital cost	Assumed
Operation and maintenance	Per annum	41	$/kW	^60^
Lead-acid battery (12 V/30Ah)				
Capital cost		450	$/kW	^61^
Installation		5	% of capital cost	Assumed
Replacement (5 years life span)		3	Times of total capital cost	Calculated
Operation and maintenance	Per annum	50	$/kW	^61^
Other parameters				
Discount rate, r_d_	Per annum (2024)	1.2	%	^62^
Lifetime, N		20	Years	Assumed
Exchange rates	Pound/USD	1.23		
	USD/JYEN	155.8		

### Study limitations

This study presents simulation results and LCOE analysis of PV/T-based microgrids with improved performance and economic viability insights, respectively. However, several limitations were encountered such as: (i) integration of the thermal and electrical models for performance optimization which greatly relies on standardized component design specifications and material properties, which are in turn not readily available from the growing technology, (ii) The assumptions used for the thermal model development neglected some aspects like heat capacity effects with isothermal temperature distribution and considered uniform heat transfer, which can lead to mismatches, and (iii) The lack of an experimental system to validate the PV/T system for long periods to assess reliability and degradation.

It is notable that, research on PV/T systems is progressing steadily due to a lack of knowledge regarding innovative cooling techniques, such as phase change materials, which are vital for enhancing efficiency and reducing the cost of the system^63^. These factors directly affect the applicability of study results in real-world scenarios. The scalability issue arises from the absence of a dedicated test setup to validate and practically assess the synergy benefits of PV/T systems. The economic analysis also indicates that energy costs for the PV/T-based microgrid are relatively high. This is primarily due to significant upfront investments and long-term maintenance expenses, which pose a barrier to achieving substantial savings. Additionally, while the study demonstrates improved performance, its applicability relies on adequate regulatory support.

## Conclusion

In this study, a PV/T system was optimally developed with a thermal-electrical model based on maximum power generation to find the operational parameters usually not specified by manufacturers. An optimal flow rate of 0.0028 kg/s per tube was obtained based on the meteorological conditions of Fukuoka, Japan. The PV/T component was then developed and integrated with the wind turbine system/battery/dynamic load using the obtained design parameters. A 3-day performance evaluation based on defined scenarios (rainy, windy, cloudy, and sunny) was performed to compare the power enhancement between the PV/T/wind/battery microgrid and the conventional PV/wind//battery microgrid. The results revealed that the extra-cumulative electricity generation from the PV/T system-based microgrid, when compared to the PV system-based microgrid, was 2.12, 2.74, 1.72, and 0.31% for the cloudy, rainy, sunny, and windy scenarios, respectively.

Regardless of promising results, active operation of the cooling system, absorber design, and cooling medium management may even increase electrical output. As such, in the present study, the effects of drawing water from the system are ignored. Usually, there will be a cooling phase where the water reaches a temperature at which point the PV/T module won’t be cooled, and its electrical efficiency will either match or fall short of that of the PV system. Therefore, control of water discharge from the system can be considered for more heat extraction. Nevertheless, a positive change in SoC bounded at about 0.005%, equivalent to 1.8 Wh for the considered simulation duration, was realized. This signifies improved system operation due to reduced deep discharge. Generally, the approach of combining two system models (electrical and thermal) through optimization and a dynamic simulation considering system state dynamics qualitatively offers a workable way of improving system effectiveness in microgrids. This synergy renders the framework valuable in increasing the need for renewable energy solutions, which have not yet been studied. Moreover, the small-scale system can be successfully scaled to any level of application based on user demand and resource availability, which are subject to techno-economic and operational challenges. This is also immensely backed by the involvement of several stakeholders and regulations broadening applicability, deployment, and energy resilience with spillover merits such as energy savings and carbon resilience.

Since the current study lacks a robust experimental system to validate the developed model, future works will focus on developing the presented PV/T system and its integration with an existing indoor microgrid system to further conduct a reliable performance evaluation. Moreover, to support microgrid operation by maintaining the supply/demand balance, the overall microgrid dynamics can be improved with hybrid storage systems such as flywheels and supercapacitors. Additionally, other factors, such as economic and environmental impacts, can be included in the optimization problem to create a more optimal system. It is also essential to assess the impact of end-user requirements on the operational performance of the proposed microgrid. This can be addressed through the implementation of demand response programs that align customers’ load profiles with microgrid reliability during critical time periods.

## Electronic supplementary material

Below is the link to the electronic supplementary material.

Supplementary Material 1

## Data Availability

All data used and analyzed in this study are provided on request from the corresponding author.

## List of symbols

1D: One dimensional
2D: Two dimensional
3D: Three dimensional
$a$: Modified diode ideality factor
$$a-Si$$: Amorphous silicon
$$AC$$: Alternating current
$AGM$: Absorbent glass mat
$ARC$: Anti-reflective coating
$c$: Specific heat capacity (J/kgK)
$$c-Si$$: Monocrystalline silicon
$${C}^{*}$$: Conductance (S)
$COP$: Coefficient of performance
$CPV/T$: Concentration photovoltaic thermal
$${C}_{\text{fixed}}$$: Fixed costs
$${C}_{op-n}$$: Operating costs
$$D$$: Diameter (m)
$$DC$$: Direct current
$DoD$: Depth of discharge
$$eV$$: Electron volt
$$E$$: Efficiency
${E}_{g}$: Silicon bandgap energy (eV)
$EVA$: Ethyl vinyl acetate
${E}_{tot-n}$: Total annual energy generation
$$G$$: Solar irradiation (W/m^2^)
$GAMS$: General algebraic modeling system
${h}_{1}$: Heat transfer coefficient from the glass panel to ambient by convection (W/m^2^K)
${h}_{2}$: Heat transfer coefficient from the glass panel to the sky by radiation (W/m^2^K)
${h}_{3}$: Heat transfer coefficient from the PV to absorber by conduction (W/m^2^K)
${h}_{4}$: Heat transfer coefficient from the PV to tube by conduction (W/m^2^K)
${h}_{5}$: Heat transfer coefficient from the absorber to tube by conduction (W/m^2^K)
${h}_{6}$: Heat transfer coefficient from the absorber to insulation by convection (W/m^2^K)
${h}_{7}$: Heat transfer coefficient from the tube to the fluid by convection (W/m^2^K)
${h}_{8}$: Heat transfer coefficient from the tube to insulation by conduction (W/m^2^K)
${h}_{9}$: Heat transfer coefficient from the tube to the fluid by convection (W/m^2^K)
${h}_{10}$: Heat transfer coefficient from the insulation to ambient by convection (W/m^2^K)
$HRES$: Hybrid renewable energy system
$I$: PV output current (A)
$${I}_{L}$$: Photocurrent (A)
${I}_{mp}$: Current at maximum power point (A)
${I}_{o}$: Diode saturation current (A)
${I}_{sc}$: Short circuit current (A)
$IGBT$: Insulated gate bipolar transistor
$K$: Polarization constant (V/Ah) or the polarization resistance ($\Omega$)
$$L$$: Channel length (m)
${L}_{i}$: Length of connecting tubes (m)
$\dot{m}$: Mass flowrate per tube (kg/s)
${\dot{m}}_{l}$: Draw off flowrate (kg/s)
$MPPT$: Maximum Power Point Tracking
$$n$$: Risers/pipes
${n}^{d}$: Diode ideality factor
$$N$$: Time (h)
${N}_{s}$: Number of PV cells
$$pc-Si$$: Polycrystalline silicon
$$P$$: Power (W)
$PCM$: Phase Change Materials
$$PF$$: Packing factor
$P\&O$: Perturb and observe
$$PV$$: Photovoltaic
$$PV/T$$: Photovoltaic thermal
$PWM$: Pulse width modulation
$$q$$: Electron charge (C)
$Q$: Total energy flux (W)
${Q}_{1}$: Total energy flux absorbed by the PV through the glass panel (W)
${Q}_{2}$: Total energy flux from the glass panel to ambient by convection (W)
${Q}_{3}$: Total energy flux from the glass panel to the sky by radiation (W)
${Q}_{4}$: Total energy flux from the PV to absorber by conduction (W)
${Q}_{5}$: Total energy flux from the PV to the tube by conduction (W)
${Q}_{6}$: Total energy flux converted into electrical (W)
${Q}_{7}$: Total energy flux from the absorber to the tube by conduction (W)
${Q}_{8}$: Total energy flux from the absorber to insulation by conduction (W)
${Q}_{9}$: Total energy flux from the tube to the fluid by convection (W)
${Q}_{10}$: Total energy flux from tube to insulation by conduction (W)
${Q}_{11}$: Total energy flux from insulation to ambient by convection (W)
${Q}_{12}$: Total energy flux absorbed by fluid (W)
${Q}_{13}$: Total energy flux from collector to tank through the spiral heat exchanger by convection (W)
${Q}_{14}$: Total energy flux from tank to ambient (W)
${Q}_{15}$: Total user thermal demand (W)
$${r}_{d}$$: Discount rate (%)
$$Re$$: Reynolds number
$${R}_{s}$$: Series resistance ($$\Omega$$)
$${R}_{\text{sh}}$$: Shunt resistance ($$\Omega$$)
$SA$: Inner surface area of the tank (m^2^)
$SoC$: State of charge (%)
$STC$: Solar thermal collector
$t$: Insulation thickness for applicable fluid temperature and pipe size (mm)
$$T$$: Temperature (K)
$U$: Heat loss coefficient (W/m^2^K)
$$UV$$: Ultraviolet
$v$: Average velocity/speed (m/s)
$$V$$: Voltage* (V)*
${V}_{mp}$: Voltage at maximum power point (V)
${V}_{oc}$: Open circuit voltage (V)
$$w$$: Channel spacing (m)
$$x$$: Thickness (m)

## Subscripts

$$a$$: Ambient/air
$$abs$$: Absorber
$$ad$$: Adhesive
$$bo$$: Bond
$$c$$: Cell
$$co{\text{-}}in$$: Collector inlet
$$co{\text{-}}out$$: Collector outlet
$$ele$$: Electrical
$$f$$: Mean fluid
$$g$$: Glass
$$h$$: Hydraulic
$$i$$: Inner
$$ins$$: Insulation
$$o$$: Outer
$$ref$$: Reference
$$req$$: Required
$$s$$: Sky
$S$: Absorbed solar irradiation (W/m^2^)
$STC$: Standard test condition
$$th$$: Thermal
$$t$$: Tube
$$tank$$: Tank
$$w$$: Wind

## Greek symbols

$$\alpha$$: Absorptivity
$$\tau$$: Transmittance
$\sigma$: Stefan Boltzmann constant (W/m^2^K^4^)
$\alpha \tau$: Product of effective absorptivity and transmissivity
${\beta }_{c}$: Power temperature coefficient (%/K)
$$\varepsilon$$: Emissivity
${\varepsilon }_{H}$: Heat exchanger effectiveness
$k$: Thermal conductivity (W/mK)
$${k}^{b}$$: Boltzmann constant (J/K)
$$\eta$$: Efficiency
$$\rho$$: Density (kg/m^3^)
$\mu$: Dynamic viscosity (kg/ms)
${\mu }_{L,sc}$: Short circuit current temperature coefficient (%/K)
