# Supplementary material for: Modeling and performance evaluation of hybrid photovoltaic thermal, wind, and battery microgrids using optimization and dynamic simulation
Source: Sci Rep. 2025 Apr 4;15:11528. doi: 10.1038/s41598-025-95149-w (PMC11971452; doi:10.1038/s41598-025-95149-w)
Supplement: Supplementary file 1 — Supplementary Material 1 [file 41598_2025_95149_MOESM1_ESM.docx]

**Supplementary material for:**

**Modeling and performance evaluation of hybrid Photovoltaic Thermal, wind, and battery microgrids using optimization and dynamic simulation**

Etoju Jacob^1^ and Hooman Farzaneh^1,2,^*

*^1^Interdisciplinary Graduate School of Engineering Sciences, Kyushu University, Fukuoka 816-8580, Japan*

*^2^ Transdisciplinary Research and Education Center for Green Technologies, Kyushu University, Fukuoka, Japan*

*Correspondence: [farzaneh.hooman.961@m.kyushu-u.ac.jp](mailto:farzaneh.hooman.961@m.kyushu-u.ac.jp)

# **S.1. PV/T electrical model parameter extraction.**

The output current of the PV module is given as ^1^;

$$I=I_{L}-I_{D}-I_{\mathrm{sh}}=I_{L}-I_{o}\left[ \exp\left( \frac{V+IR_{s}}{a} \right)-1 \right]-\frac{V+IR_{s}}{R_{\mathrm{sh}}}$$

1. **Parameter initialization at reference conditions (standard test conditions).**

$a_{ref}=\frac{n^{d}k^{b}T_{c, ref}N_{s}}{q}$  *(n assumed 1.5)* (S.1)

${I_{sc,ref}=I}_{sc}$ (S.2)

${I_{L,ref}=I}_{sc,ref}$ (S.3)

${V_{oc,ref}=V}_{oc}$ (S.4)

$I_{o,ref}=I_{sc,ref} exp \left( \frac{-V_{oc,ref}}{a_{ref}} \right)$ (S.5)

$I_{mp,ref}=I_{mp}$ (S.6)

$V_{mp,ref}=V_{mp}$ (S.7)

$R_{s,ref}= \frac{a_{ref}\ln\left[ \frac{I_{sc,ref}-I_{mp,ref}}{I_{o,ref}} + 1 \right] - V_{mp,ref}}{I_{mp,ref}}$ (S.8)

$R_{sh,ref}= 100 \Omega$ *(initial value)*  (S.9)

1. **Solar cell parameter extraction** ($I_{L}, I_{o}, R_{s}, R_{sh}, a )$.

Formulations are made based on the I-V and P-V curve characteristics and solved numerically to determine the five parameters.

1. At short circuit ($V=0, I=I_{sc,\mathrm{ref}}$).

$I_{L}-I_{o}\left[ exp\left( \frac{I_{sc}R_{s}}{a} \right)-1 \right]-\frac{I_{sc}R_{s}}{R_{sh}}-I_{sc} =0$ (S.10)

1. At open circuit ($V=V_{oc,ref}, I=0$ ).

$I_{o}\left[ exp\left( \frac{V_{oc}}{a} \right)-1 \right]+\frac{V_{oc}}{R_{sh}}-I_{L}=0$ (S.11)

1. At maximum power point ($V=V_{mp}, I=I_{mp}$).

$I_{L}-I_{o}\left[ exp\left( \frac{V_{mp}+I_{mp}R_{s}}{a} \right)-1 \right]-\frac{V_{mp}+I_{mp}R_{s}}{R_{sh}}-I_{mp} =0$ (S.12)

1. At maximum power point ( $\frac{dP}{dV}=0$).

$V_{mp}\left[ \frac{\frac{I_{o}}{a}exp\left( \frac{V_{mp}+I_{mp}R_{s}}{a} \right)+\frac{1}{R_{sh}}}{1 + \frac{R_{s}}{R_{sh}} + \frac{I_{o}R_{s}}{a}exp\left( \frac{V_{mp}+I_{mp}R_{s}}{a} \right)} \right]-I_{mp}=0$ (S.13)

1. At maximum power point ( $\frac{dP}{dI}=0$).

$I_{mp}\left[ \frac{1 + \frac{R_{s}}{R_{sh}} + \frac{I_{o}R_{s}}{a}exp\left( \frac{V_{mp}+I_{mp}R_{s}}{a} \right)}{\frac{I_{o}}{a}exp\left( \frac{V_{mp}+I_{mp}R_{s}}{a} \right)+\frac{1}{R_{sh}}} \right]-V_{mp}=0$ (S.14)

# **S.2. CONOPT and the Generalized Reduced Gradient (GRG) algorithm details.**

The details of the GRG algorithm are presented below.

Firstly, CONOPT can take advantage of dynamic models but for simplicity, the time independent general NLP problem is considered as ^2,3^:

$\left\{ \begin{aligned} max/min f(X) \\ s.t \\ C_{i}\left( X \right)=b_{i} ; i=1,\ldots,m \\ l_{i}\leq X_{i}\leq u_{i} ; i=1,\ldots,n \end{aligned} \right.$ (S.15)

where: $X\in\mathbb{R}^{n}$ (is an *n*-dimensional vector of optimization variables), $f:\mathbb{R}^{n}\to\mathbb{R}$, $C_{i}:\mathbb{R}^{n}\to\mathbb{R}^{m}$ (is a mapping of $\mathbb{R}^{n}\to\mathbb{R}^{m}$), $b_{i}\in\mathbb{R}^{n}$ (is an *m*-dimensional vector of the right hand side), $l_{i} \& u_{i}{\in\mathbb{R}}^{n}$ (n vectors of lower and upper variable bounds) ^4^.

The inequality constraints in equation (S.15) are transformed into equalities by addition of properly bounded slack variables. Hence vector *X* contains both original optimization variables and slacks. This implies that the Jacobian of constraints includes a unit matrix and hence has a full row rank ^4^.

In CONOPT, the basic idea of GRG is to use the equalities in equation (S.15) to express *m* of the variables as basic variables in terms of the rest *n-m* (non-basic variables) ^3^. Vector *X* is subdivided into $\hat{X}\in\mathbb{R}^{n}$ (basic variables) and $\tilde{X}\in\mathbb{R}^{n-m}$ (non-basic variables). Therefore, the system of nonlinear constraints $C_{i}\left( X \right)=b_{i}$ can be rewritten as $\hat{X}=C_{1}(\tilde{X})$ ^4^.

**Definition:**

From the implicit function theorem, let $X_{o}$ be an arbitrary feasible solution. Then, $X_{o}=(\hat{X}_{o},\tilde{X}_{o})$ satisfies $C\left( \hat{X}_{o},\tilde{X}_{o} \right)=0$ and the Jacobian, $\frac{\partial C}{\partial\hat{X}}$ has rank *m*. Hence for solutions close to $X_{o}$, it is possible to transform $C\left( \hat{X},\tilde{X} \right)=0$ into $\hat{X}=C_{1}(\tilde{X})$. The function $C_{1}$ is differentiable and its Jacobian is found by implicit differentiation as:

$\frac{\partial C}{\partial X}dX=\frac{\partial C}{\partial\hat{X}}d\hat{X}+\frac{\partial C}{\partial\tilde{X}}d\tilde{X}=0$ (S.16)

From equation (S.16),

$\frac{\partial C_{1}}{\partial\tilde{X}}=\frac{\partial\hat{X}}{\partial\tilde{X}}=-\left( \frac{\partial C}{\partial\hat{X}} \right)^{-1}\frac{\partial C}{\partial\tilde{X}}$ (S.17)

The basic variables are determined by solving the system $C\left( \hat{X},\tilde{X} \right)=0$ iteratively. The equation $\hat{X}=C_{1}(\tilde{X})$ is then introduced into equation (S.15) as:

$F\left( \tilde{X} \right)\triangleq f\left( C_{1}\left( \tilde{X} \right),\tilde{X} \right)$ (S.18)

Using equation (S.17) with (S.18), $\frac{\partial F}{\partial\tilde{X}}=\frac{\partial f}{\partial\hat{X}}\frac{\partial\hat{X}}{\partial\tilde{X}}+\frac{\partial f}{\partial\tilde{X}}=\frac{\partial f}{\partial\tilde{X}}-\frac{\partial f}{\partial\hat{X}}\left( \frac{\partial C}{\partial\hat{X}} \right)^{-1}\frac{\partial C}{\partial\tilde{X}}$ , this derivative is the **reduced gradient** depicting the influence on the objective function of changes in non-basic variables considering basic variables ^4^. Hence equation (S.15) is transformed into a reduced problem equation (S.19), with only upper and lower bounds which the GRG algorithm solves.

$\left\{ \begin{aligned} max/min F(\tilde{X}) \\ s.t \\ \hat{X}=C_{1}(\tilde{X}) \\ \left( \hat{l,}\tilde{l} \right)\leq\left( \hat{X},\tilde{X} \right)\leq(\hat{u},\tilde{u}) \end{aligned} \right.$ (S.19)

# **S.3. PV/T user-defined mask component.**

The component inputs are irradiation-G (*W/m^2^*), wind velocity-Vw (*m/s*), and ambient temperature-Ta (֯*C*), while the outputs include voltage(*V*), current (*A*), cell temperature (*K*), electrical and thermal efficiency through m as shown in Figure S. 1.(a). When integrated into the simulation model, computation time was a major drawback due to the concurrent calling of an extrinsic function for each time step. Alternatively, the PV/T system was realized with a 3-D lookup table depicted in Figure S. 1. (b).


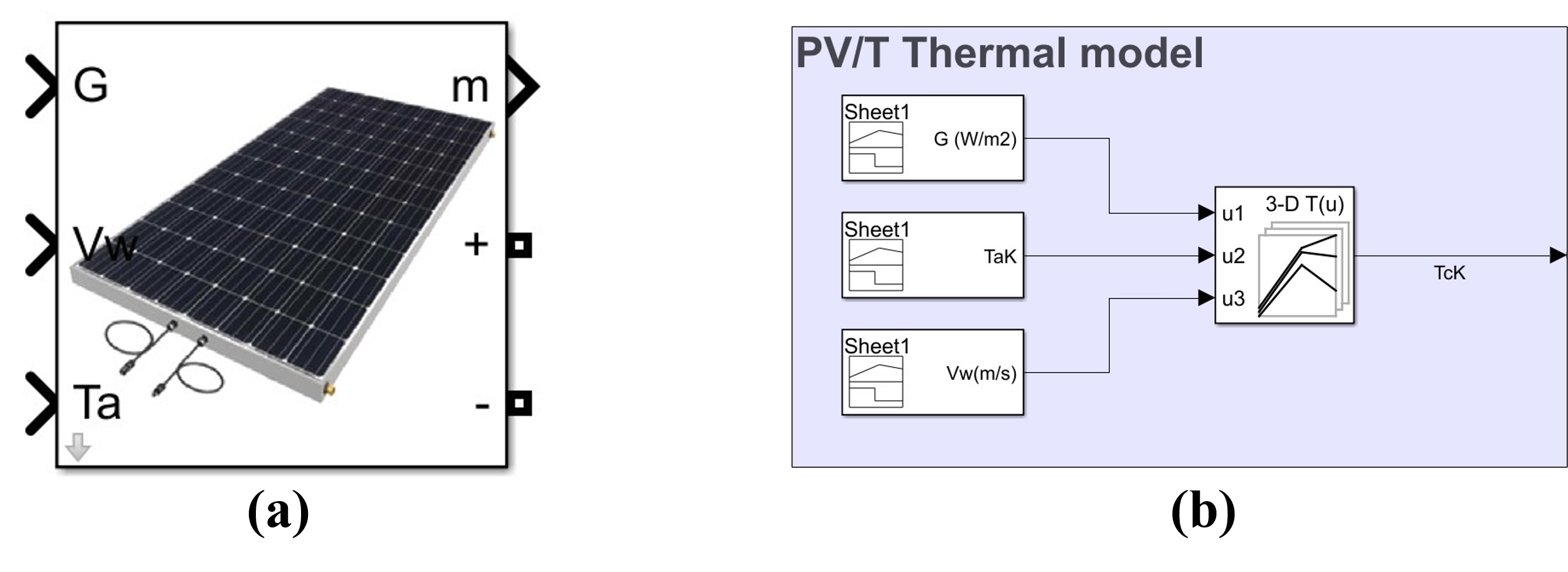


Figure S. 1. (a) Developed PV/T mask component, (b) PV/T model with a 3-D Lookup table

# **S.4. Sample meteorological data extract:**

The weather data from Japan Meteorological Agency is classified based on the weather indicator and cloud cover in the last two columns as shown in Figure S. 2^5^.

Figure S. 2. Sample data extract for Fukuoka-Japan on 12^th^ July ,2021 from 5 to 18 hours

In Figure S. 3, the cloud cover indicator is explained. It ranges from 0+ to 10 increasing with order of magnitude. **Note:** The + and – is only for those values indicated.ie. (0+, 1, 2, 3, 4, 5, 6, 7, 8, 9, 10-, 10).

Figure S. 3. Cloud cover Indicator

The weather indicators that describe the hourly conditions are described in Figure S. 4. It should be noted that when the data is downloaded, the CSV file only shows the weather symbol value.

*In visual observations, if there is more than one applicable weather value 1-15, the one with the higher value is given priority.

Figure S. 4. Weather Indicators

# **S.5. Hourly generation (*Wh*) from the microgrid with PV/T and that with PV.**

Table S. 1. hourly generation from PV and PV/T

|  | Cloudy | | Rainy | | Sunny | | Windy | |
| --- | --- | --- | --- | --- | --- | --- | --- | --- |
| Time (Hr) | **PV** | **PV/T** | **PV** | **PV/T** | **PV** | **PV/T** | **PV** | **PV/T** |
| 1 | 0 | 0 | 0 | 0 | 0 | 0 | 0 | 0 |
| 2 | 0 | 0 | 0 | 0 | 0 | 0 | 0 | 0 |
| 3 | 0 | 0 | 0 | 0 | 0 | 0 | 0 | 0 |
| 4 | 0 | 0 | 0 | 0 | 0 | 0 | 0 | 0 |
| 5 | 0 | 0 | 0 | 0 | 0 | 0 | 0 | 0 |
| 6 | 0 | 0 | 0.465 | 0.465 | 2.349 | 2.357 | 0 | 0 |
| 7 | 13.316 | 13.883 | 6.023 | 6.168 | 27.331 | 28.668 | 3.028 | 3.069 |
| 8 | 25.288 | 26.423 | 8.358 | 7.974 | 72.778 | 79.975 | 21.591 | 21.326 |
| 9 | 55.045 | 59.756 | 9.075 | 8.624 | 119.925 | 127.669 | 29.527 | 32.52 |
| 10 | 69.345 | 74.588 | 9.827 | 9.24 | 168.14 | 170.797 | 25.29 | 25.286 |
| 11 | 98.858 | 104.716 | 9.842 | 9.245 | 200.681 | 203.872 | 30.559 | 32.087 |
| 12 | 156.936 | 159.732 | 10.813 | 10.995 | 213.628 | 217.389 | 23.857 | 24.431 |
| 13 | 68.467 | 71.587 | 8.566 | 8.546 | 218.637 | 219.107 | 21.638 | 21.949 |
| 14 | 128.14 | 128.551 | 6.494 | 6.487 | 210.176 | 211.152 | 6.508 | 6.578 |
| 15 | 70.446 | 74.346 | 5.788 | 5.786 | 189.942 | 191.144 | 5.04 | 5.081 |
| 16 | 77.207 | 78.922 | 7.111 | 7.109 | 156.579 | 158.895 | 5.005 | 5.059 |
| 17 | 67.151 | 70.316 | 8.476 | 8.458 | 114.159 | 119.782 | 2.39 | 2.452 |
| 18 | 28.436 | 27.898 | 11.777 | 12.234 | 73.608 | 69.753 | 1.777 | 1.807 |
| 19 | 7.055 | 7.247 | 6.358 | 6.389 | 22.56 | 22.708 | 0.01 | 0.01 |
| 20 | 0.04 | 0.041 | 0.038 | 0.038 | 0.721 | 0.733 | 0 | 0 |
| 21 | 0 | 0 | 0 | 0 | 0.003 | 0.003 | 0 | 0 |
| 22 | 0 | 0 | 0 | 0 | 0 | 0 | 0 | 0 |
| 23 | 0 | 0 | 0 | 0 | 0 | 0 | 0 | 0 |
| 24 | 0 | 0 | 0 | 0 | 0 | 0 | 0 | 0 |
| 25 | 0 | 0 | 0 | 0 | 0 | 0 | 0 | 0 |
| 26 | 0 | 0 | 0 | 0 | 0 | 0 | 0 | 0 |
| 27 | 0 | 0 | 0 | 0 | 0 | 0 | 0 | 0 |
| 28 | 0 | 0 | 0 | 0 | 0 | 0 | 0 | 0 |
| 29 | 0 | 0 | 0 | 0 | 0 | 0 | 0 | 0 |
| 30 | 0 | 0 | 0 | 0 | 1.637 | 1.655 | 0 | 0 |
| 31 | 1.599 | 1.623 | 1.664 | 1.69 | 26.755 | 26.347 | 1.099 | 1.102 |
| 32 | 8.869 | 9.01 | 5.656 | 5.48 | 57.955 | 59.004 | 5.728 | 5.733 |
| 33 | 18.036 | 19.026 | 2.454 | 2.478 | 114.893 | 115.847 | 31.172 | 31.671 |
| 34 | 52.009 | 54.788 | 2.431 | 2.449 | 166.885 | 173.093 | 62.125 | 64.273 |
| 35 | 45.377 | 50.122 | 8.529 | 8.399 | 195.338 | 200.555 | 128.119 | 129.932 |
| 36 | 52.753 | 55.751 | 15.701 | 15.573 | 214.209 | 216.46 | 131.621 | 131.562 |
| 37 | 136.818 | 137.385 | 17.109 | 17.806 | 221.243 | 222.263 | 189.325 | 189.298 |
| 38 | 114.93 | 119.618 | 27.437 | 27.27 | 215.098 | 214.006 | 202.648 | 199.12 |
| 39 | 132.526 | 131.171 | 31.464 | 34.035 | 192.585 | 194.975 | 135.192 | 135.286 |
| 40 | 79.143 | 81.88 | 23.905 | 24.884 | 155.77 | 157.494 | 101.82 | 102.183 |
| 41 | 39.346 | 39.817 | 21.112 | 21.869 | 111.675 | 115.818 | 56.77 | 55.151 |
| 42 | 21.859 | 22.114 | 8.824 | 8.807 | 63.433 | 65.361 | 22.083 | 22.051 |
| 43 | 1.724 | 1.819 | 1.859 | 1.881 | 19.251 | 19.835 | 1.345 | 1.35 |
| 44 | 0.01 | 0.01 | 0.01 | 0.01 | 0.702 | 0.714 | 0.007 | 0.007 |
| 45 | 0 | 0 | 0 | 0 | 0.003 | 0.003 | 0 | 0 |
| 46 | 0 | 0 | 0 | 0 | 0 | 0 | 0 | 0 |
| 47 | 0 | 0 | 0 | 0 | 0 | 0 | 0 | 0 |
| 48 | 0 | 0 | 0 | 0 | 0 | 0 | 0 | 0 |
| 49 | 0 | 0 | 0 | 0 | 0 | 0 | 0 | 0 |
| 50 | 0 | 0 | 0 | 0 | 0 | 0 | 0 | 0 |
| 51 | 0 | 0 | 0 | 0 | 0 | 0 | 0 | 0 |
| 52 | 0 | 0 | 0 | 0 | 0 | 0 | 0 | 0 |
| 53 | 0 | 0 | 0 | 0 | 0 | 0 | 0 | 0 |
| 54 | 0 | 0 | 0 | 0 | 1.638 | 1.648 | 0 | 0 |
| 55 | 3.613 | 3.695 | 6.204 | 5.875 | 24.532 | 24.053 | 7.033 | 7.047 |
| 56 | 23.994 | 24.57 | 14.883 | 15.101 | 66.659 | 68.255 | 32.764 | 32.627 |
| 57 | 65.873 | 68.298 | 28.546 | 30.778 | 121.291 | 118.9 | 102.172 | 102.795 |
| 58 | 77.527 | 80.397 | 39.509 | 44.908 | 160.503 | 169.888 | 99.101 | 99.906 |
| 59 | 133.854 | 133.947 | 25.049 | 25.847 | 189.938 | 197.637 | 134.368 | 134.992 |
| 60 | 170.925 | 173.002 | 29.749 | 31.503 | 211.541 | 215.151 | 117.85 | 117.41 |
| 61 | 203.231 | 207.056 | 15.136 | 15.266 | 219.658 | 220.653 | 133.63 | 133.325 |
| 62 | 203.144 | 205.606 | 13.746 | 13.776 | 212.244 | 212.265 | 93.609 | 93.999 |
| 63 | 120.346 | 121.884 | 10.508 | 10.615 | 193.872 | 193.533 | 152.226 | 151.625 |
| 64 | 118.387 | 117.544 | 5.133 | 5.077 | 151.25 | 156.422 | 104.889 | 105.497 |
| 65 | 88.294 | 84.283 | 1.854 | 1.859 | 111.006 | 115.382 | 43.426 | 43.33 |
| 66 | 39.945 | 37.111 | 2.415 | 2.428 | 65.638 | 67.445 | 19.316 | 19.374 |
| 67 | 6.438 | 6.477 | 3.731 | 3.751 | 17.265 | 17.863 | 0.707 | 0.712 |
| 68 | 0.036 | 0.036 | 0.02 | 0.02 | 0.684 | 0.697 | 0.003 | 0.003 |
| 69 | 0 | 0 | 0 | 0 | 0.003 | 0.003 | 0 | 0 |
| 70 | 0 | 0 | 0 | 0 | 0 | 0 | 0 | 0 |
| 71 | 0 | 0 | 0 | 0 | 0 | 0 | 0 | 0 |
| 72 | 0 | 0 | 0 | 0 | 0 | 0 | 0 | 0 |

# **References:**

1. Duffie, J. A., Beckman, W. A. & McGowan, J. *Solar Engineering of Thermal Processes*. *American Journal of Physics* vol. 53 (1985).

2. Drud, A. CONOPT: A GRG code for large sparse dynamic nonlinear optimization problems. *Math. Program.* **31**, 153–191 (1985).

3. Lasdon, L. S., Fox, R. L. & Ratner, M. W. Nonlinear optimization using the generalized reduced gradient method.

4. Andrei, N. Continuous Nonlinear Optimization for Engineering Applications in GAMS Technology. **121**, (2017).

5. Japan Meteorological Agency. https://www.data.jma.go.jp/risk/obsdl/index.php#.
